# RTN4IP1 Contributes to ESCC via Regulation of Amino Acid Transporters

**DOI:** 10.1002/advs.202406220

**Published:** 2025-01-05

**Authors:** Huifang Wei, Dengyun Zhao, Yafei Zhi, Qiong Wu, Jing Ma, Jialuo Xu, Tingting Liu, Jing Zhang, Penglei Wang, Yamei Hu, Xinyu He, Fangqin Guo, Ming Jiang, Dandan Zhang, Wenna Nie, Ran Yang, Tongjin Zhao, Zigang Dong, Kangdong Liu

**Affiliations:** ^1^ Department of Pathophysiology School of Basic Medical Sciences Zhengzhou University, China‐US (Henan) Hormel Cancer Institute Zhengzhou 450000 China; ^2^ Department of Pathophysiology School of Basic Medical Sciences, Zhengzhou University China‐US (Henan) Hormel Cancer Institute Chest Hospital of Zhengzhou University Zhengzhou 450000 China; ^3^ Department of Pathophysiology School of Basic Medical Sciences Zhengzhou University Zhengzhou 450000 China; ^4^ China‐US (Henan) Hormel Cancer Institute Zhengzhou 450000 China; ^5^ Department of Pathophysiology School of Basic Medical Sciences Tianjian Laboratory of Advanced Biomedical Sciences Zhengzhou University Zhengzhou 450000 China; ^6^ State Key Laboratory of Genetic Engineering Shanghai Key Laboratory of Metabolic Remodeling and Health Institute of Metabolism and Integrative Biology Zhongshan Hospital Shanghai Qi Zhi Institute Fudan University Shanghai 200438 China; ^7^ Department of Pathophysiology School of Basic Medical Sciences The Collaborative Innovation Center of Henan Province for Cancer Chemoprevention, State Key Laboratory of Esophageal Cancer Prevention and Treatment Provincial Cooperative Innovation Center for Cancer Chemoprevention China‐US (Henan) Hormel Cancer Institute, Tianjian Laboratory of Advanced Biomedical Sciences Zhengzhou University Zhengzhou 450000 China

**Keywords:** Amino acid transporters, c‐Myc, ESCC, Iron regulatory proteins, Iron responsive element, RTN4IP1

## Abstract

Esophageal squamous cell carcinoma (ESCC) accounts for about 90% of esophageal cancer cases. The lack of effective therapeutic targets makes it difficult to improve the overall survival of patients with ESCC. Reticulon 4 Interacting Protein 1 (RTN4IP1) is a novel mitochondrial oxidoreductase. Here, a notable upregulation of RTN4IP1 is demonstrated, which is associated with poor survival in patients with ESCC. RTN4IP1 depletion impairs cell proliferation and induces apoptosis of ESCC cells. Furthermore, c‐Myc regulates RTN4IP1 expression via iron regulatory protein 2 (IRP2) at the post‐transcriptional level. Mechanistically, *RTN4IP1* mRNA harbors functional iron‐responsive elements (IREs) in the 3′ UTR, which can be targeted by IRP2, resulting in increased mRNA stability. Finally, RTN4IP1 depletion abrogates amino acid uptake and induces amino acid starvation via downregulation of the amino acid transporters SLC1A5, SLC3A2, and SLC7A5, indicating a possible pathway through which RTN4IP1 contributes to ESCC carcinogenesis and progression. In vivo studies using cell‐derived xenograft and patient‐derived xenograft mouse models as well as a 4‐nitroquinoline 1‐oxide‐induced ESCC model in esophageal‐specific *Rtn4ip1* knockout mice demonstrate the essential role of RTN4IP1 in ESCC development. Thus, RTN4IP1 emerges as a key cancer‐promoting protein in ESCC, suggesting therapeutic RTN4IP1 suppression as a promising strategy for ESCC treatment.

## Graphical abstract

RTN4IP1 is a putative mitochondrial oxidoreductase with unknown biological functions in cancer. The present study revealed that RTN4IP1 functions as a cancer‐promoting protein in ESCC by regulating amino acid transporters. The c‐Myc–IRP2–IRE pathway is responsible for the elevated expression of RTN4IP1 via modulation of mRNA decay. Therefore, targeting RTN4IP1 may be a promising strategy for ESCC treatment.

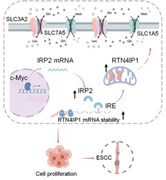

## 1 Introduction

Esophageal cancer is one of the most commonly diagnosed malignancies and the sixth leading cause of cancer‐related death worldwide.^[^ ^1^ ^]^ Esophageal squamous cell carcinoma (ESCC) and esophageal adenocarcinoma (EAC) are the two main histopathological subtypes of esophageal cancer, of which ESCC accounts for ≈90% of all reported cases of esophageal cancer worldwide. Currently, the primary curative treatment for ESCC typically includes chemotherapy or chemoradiotherapy followed by extensive surgery.^[^ ^2^ ^]^ In recent years, an increasing number of studies have focused on the development of targeted therapeutic drugs against ESCC. Inhibitors of PD‐L1 (such as pembrolizumab, nivolumab, sintilimab, camrelizumab, and toripalimab), VEGFR2 (such as lapatinib), HER‐2 (such as trastuzumab) and other targets (such as anlotinib) have been verified to moderately improve survival.^[^ ^2^, ^3^, ^4^, ^5^, ^6^ ^]^ Some targeted therapeutic drugs, including pembrolizumab, nivolumab, lapatinib, and anlotinib, have been approved for the first‐ or second‐line treatment of ESCC; however, these drugs are associated with a limited duration of overall survival, which is still far from satisfactory.^[^ ^2^, ^3^, ^4^, ^5^, ^6^ ^]^ Drug resistance, limited response depending on population differences and severe adverse effects are other major challenges.^[^ ^2^ ^]^ Therefore, there remains an urgent need to better understand the molecular features of ESCC and develop novel therapies tailored to the molecular composition of ESCC.

Reticulon 4 Interacting Protein 1 (RTN4IP1) was first identified as a Nogo‐interacting protein while screening a human brain cDNA library in 2002.^[^ ^7^ ^]^ With an N‐terminal mitochondria‐targeting sequence, RTN4IP1 localizes to the mitochondria and is ubiquitously expressed in mitochondria‐enriched tissues, including heart, liver, muscle, and kidneys.^[^ ^7^ ^]^ Subsequent studies have identified a series of germline mutations in *RTN4IP1* associated with recessive optic neuropathy that may be followed by the development of additional neurological symptoms in humans, suggesting a role of RTN4IP1 in neuronal development and function.^[^ ^8^, ^9^, ^10^, ^11^, ^12^, ^13^ ^]^ Subsequently, additional details regarding RTN4IP1 have been unveiled. Park et al. verified that RTN4IP1 possesses oxidoreductase activity and plays a role in coenzyme Q (CoQ) biosynthesis. RTN4IP1 deficiency can induce mitochondrial stress and impair oxidative phosphorylation activity.^[^ ^14^ ^]^ Several studies have presented evidence for the role of RTN4IP1 in cancer. It was reported that RTN4IP1 is downregulated in thyroid cancer and exhibits tumor‐suppressive functions. RTN4IP1 knockdown promotes cell proliferation, invasion and migration, as well as colony and sphere formation in vitro.^[^ ^15^ ^]^ Van de Vijver and colleagues reported a 14‐gene expression signature specific to visceral metastasis in breast cancer, implying the involvement of RTN4IP1 in metastasis.^[^ ^16^ ^]^ Another study on germinal center B‐cell type diffuse large B‐cell lymphoma identified *RTN4IP1* as a super‐enhancer‐related and survival‐associated gene.^[^ ^17^ ^]^ However, the role of RTN4IP1 in cancer and the underlying regulatory mechanism governing its expression remain unclear.

Herein, we demonstrated that RTN4IP1 expression was substantially elevated in ESCC and defined the cancer‐promoting role of RTN4IP1 both in vitro and in vivo. We also determined that RTN4IP1 expression was positively regulated by the c‐Myc–IRP2–IRE axis at the mRNA‐stability level. Interestingly, RTN4IP1 deficiency impaired the expression of multiple amino acid transporters, resulting in amino acid starvation; thus, RTN4IP1 knockdown impaired cell proliferation and tumor growth in ESCC. These findings expand our knowledge on RTN4IP1 and will help to facilitate the development of effective therapeutic strategies against ESCC.

## 2 Results

### 2.1 RTN4IP1 is Significantly Elevated in ESCC

**Figure 1:**
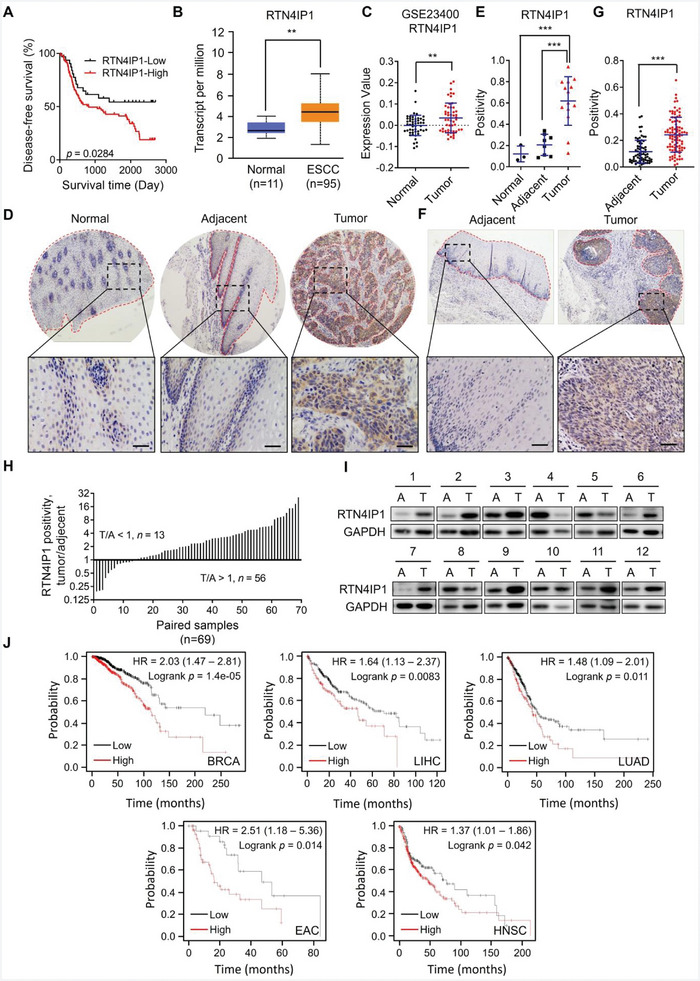
RTN4IP1 expression is elevated in ESCC. A) Graph showing disease‐free survival analysis of RTN4IP1 in ESCC proteomic study. RTN4IP1‐Low *n* = 32, RTN4IP1‐High *n* = 92. B) Graph showing mRNA expression pattern of RTN4IP1 in ESCC and normal esophageal tissues analyzed by UALCAN, *p = *0.0029. C) Graph showing analysis of microarray‐based RTN4IP1 expression, *n = *51 pairs. D) Representative IHC staining images using anti‐RTN4IP1 antibody in normal esophageal specimens (*n = *3), adjacent tissues (*n = *8) and ESCC tissues (*n = *14). Scale bar = 50 µm. E) Graph showing the analysis of IHC staining results in panel D. F) Representative IHC staining images using anti‐RTN4IP1 antibody in ESCC tissue microarray. Areas in dotted line indicate esophageal epithelium and cancer in D and F. Scale bar = 50 µm. G) Graph showing analysis of IHC staining results in panel F. Adjacent tissues, *n = *70; tumor, *n = *92. H) Graph showing the analysis of IHC staining results of paired samples (*n = *69) in panel F. Tumor/adjacent (T/A) > 1 means RTN4IP1 upregulation, < 1 means RTN4IP1 downregulation in tumors compared with paired adjacent tissues. I) Western blotting analysis of RTN4IP1 protein levels in paired ESCC and adjacent tissues. A, adjacent tissue; T, tumor tissue). J) Graphs showing overall survival analyses of RTN4IP1 by Kaplan‐Meier Plotter in different cancers. In the statistical plots, data were expressed as the mean ± SD. **, *p <* 0.01, ***, *p <* 0.001 by Student's *t*‐test (C and G) or Ordinary one‐way ANOVA (E).

We have previously revealed the essential role of chromatin reader bromodomain‐containing protein 4 (BRD4) in promoting ESCC progression.^[^ ^18^ ^]^ To identify novel metabolism‐related genes downstream of BRD4 and explore the molecular features of ESCC, we analyzed our RNA‐seq and ESCC proteomic data from a previous study.^[^ ^19^ ^]^ We found 718 downregulated genes upon BRD4 inhibitor (+)‐JQ‐1 (JQ‐1) treatment or BRD4 knockdown (BRD4‐KD) (JQ‐1/control ratio ≤ 0.5, BRD4‐KD/scramble ratio ≤ 0.5, *p <* 0.01) in the RNA‐seq data, among which protein products of 396 genes were identified via the proteomics study in ESCC. Then, we identified 273 differentially expressed proteins, including ≈30 metabolism‐related enzymes. Finally, four novel enzymes with unappreciated biological functions in cancer were identified, namely thromboxane A synthase 1 (TBXAS1), 5′,3′‐nucleotidase, cytosolic (NT5C), glycosyltransferase 8 domain containing 2 (GLT8D2) and reticulon 4 interacting protein 1 (RTN4IP1) (Figure S1A,B, Supporting Information). TBXAS1, GLT8D2 and RTN4IP1 were upregulated, whereas NT5C was downregulated in ESCC (Figure S1B, Supporting Information). Furthermore, we determined the clinical relevance of the four candidate proteins and found that high RTN4IP1 or low NT5C expression predicted poor survival in patients with ESCC (**Figure** **1**A; Figure S1C,D, Supporting Information).

Indeed, the BRD4 inhibitors JQ‐1 and OTX015 (Birabresib) blocked RTN4IP1 and NT5C expression in a dose‐dependent manner (Figure S1E,F, Supporting Information). BRD4 plays a role in cancer by promoting oncogene expression.^[^ ^20^ ^]^ Although the mRNA level of NT5C was downregulated upon genetic or pharmacological inhibition of BRD4, the expression pattern of NT5C and its clinical relevance indicated that it might be a tumor suppressor in ESCC. Moreover, only RTN4IP1 was significantly correlated with BRD4 expression in ESCC (Figure S1G, Supporting Information), suggesting that other regulators irrelevant to BRD4 may play a more important role in NT5C expression in ESCC. Moreover, our Western blotting results demonstrated that pharmacological inhibition of BRD4 decreased RTN4IP1 protein levels in ESCC cell lines (Figure S1H, Supporting Information). Therefore, we hypothesized that RTN4IP1 is a potential downstream target of BRD4.

RTN4IP1 is a mitochondrial oxidoreductase, and its function in cancer remains obscure. Consistent with the RTN4IP1 expression pattern in the ESCC proteomic study (Figure S1B, Supporting Information), subsequent analysis of RTN4IP1 expression in the TCGA database using UALCAN (http://ualcan.path.uab.edu/)^[^ ^21^ ^]^ and a microarray study of ESCC^[^ ^22^ ^]^ confirmed augmented *RTN4IP1* mRNA levels in tumors (Figure 1B,C). Further, we performed immunohistochemical staining to detect RTN4IP1 expression across a panel of primary human ESCC and adjacent tissues as well as normal esophageal tissues. RTN4IP1 staining was much stronger in ESCC tissues than in the adjacent or normal esophageal tissues (Figure 1D,E). Immunohistochemical analysis of RTN4IP1 was performed with an ESCC tissue microarray of a larger cohort. Similar to the results observed with the previous panel, RTN4IP1 staining was drastically elevated in ESCC tissues (Figure 1F,G). Moreover, RTN4IP1 protein levels were upregulated in ESCC tissues in most paired samples (Figure 1H). We validated this by assessing RTN4IP1 protein levels in paired tissues collected from patients with ESCC and found that the levels were higher in tumors (Figure 1I). Collectively, these results indicate that RTN4IP1 is upregulated in ESCC and high RTN4IP1 levels predict poor survival in patients with ESCC.

We also found that high RTN4IP1 expression correlated with poor survival in patients with other cancers, including breast cancer (BRCA), liver hepatocellular carcinoma (LIHC), lung adenocarcinoma (LUAD), esophageal adenocarcinoma (EAC), and head and neck squamous cell carcinoma (HNSC) (Figure 1J), implying that RTN4IP1 function as a contributor to multiple types of cancer.

### 2.2 RTN4IP1 Deficiency Retards ESCC Cell Proliferation and Induces Apoptosis

**Figure 2:**
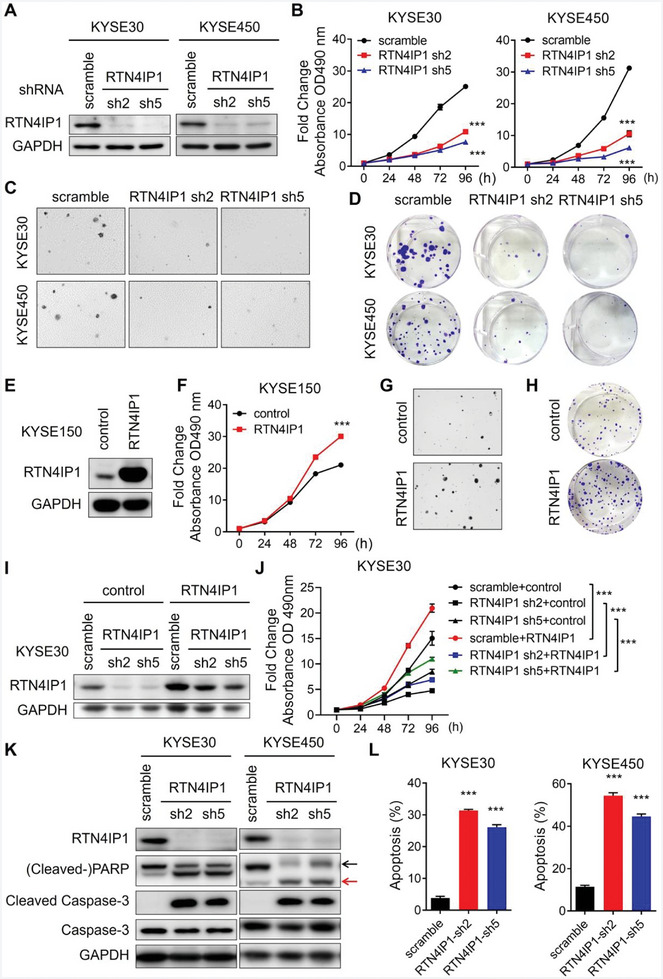
RTN4IP1 is essential to ESCC cell proliferation. A–D) KYSE30 and KYSE450 cells infected with lentivirus‐mediated shRNAs targeting RTN4IP1 or scramble were analyzed by Western blotting A), MTT assay B), soft agar assay C) and colony formation assay D). E–H) KYSE150 cell transfected with RTN4IP1 or control plasmids were analyzed by Western blotting E), MTT assay F), soft agar assay G) and colony formation assay H). I,J) KYSE30 cells infected with lentivirus‐mediated shRNAs targeting RTN4IP1 or scramble were transfected with control or RTN4IP1 plasmids, then analyzed by Western blotting I) and MTT assay J). K,L) KYSE30 and KYSE450 cells infected with lentivirus‐mediated shRNAs targeting RTN4IP1 or scramble were analyzed by Western blotting K) and flow cytometry L). The black arrow indicates pan‐PARP, red arrow indicates cleaved‐PARP. In all statistical plots, data were expressed as the mean ± SD. ***, *p <* 0.001 by Student's *t*‐test (F) or Ordinary one‐way ANOVA (B, J, and L).

Next, we investigated the role of RTN4IP1 in ESCC cell proliferation. RTN4IP1 expression was determined in different ESCC cell lines (Figure S2A, Supporting Information). We knocked down RTN4IP1 using two independent shRNAs in KYSE30 and KYSE450 cells with relatively high RTN4IP1 expression (**Figure** **2A**). Upon RTN4IP1 silencing, we observed significant impairment in cell viability, sphere formation potential, and colony‐formation ability of both cell lines compared with that in cells transfected with scramble shRNA (Figure 2B–D; Figure S2B,C, Supporting Information). In contrast, forced RTN4IP1 expression augmented the proliferative and clonogenic capacities of KYSE150 and KYSE30 cells (Figure 2E–H; Figure S2D–H, Supporting Information). We further confirmed that proliferation inhibition resulting from RTN4IP1 knockdown was efficiently rescued by RTN4IP1 re‐expression (Figure 2I,J). In addition, significant cell death was observed in conditions of RTN4IP1 deficiency, which was inhibited by Caspase inhibitors (Figure 2K,L; Figure S2I, Supporting Information), indicating that RTN4IP1 silencing induces apoptosis in ESCC cells. We also examined the effect of RTN4IP1 deficiency on cell cycle progression. EdU (5‐ethynyl‐2‐deoxyuridine) incorporation and cell cycle assays demonstrated that RTN4IP1 knockdown impaired DNA synthesis and caused cell cycle arrest (Figure S2J,K, Supporting Information).

### 2.3 c‐Myc Regulates RTN4IP1 Expression in a Transcription‐Independent Manner

**Figure 3:**
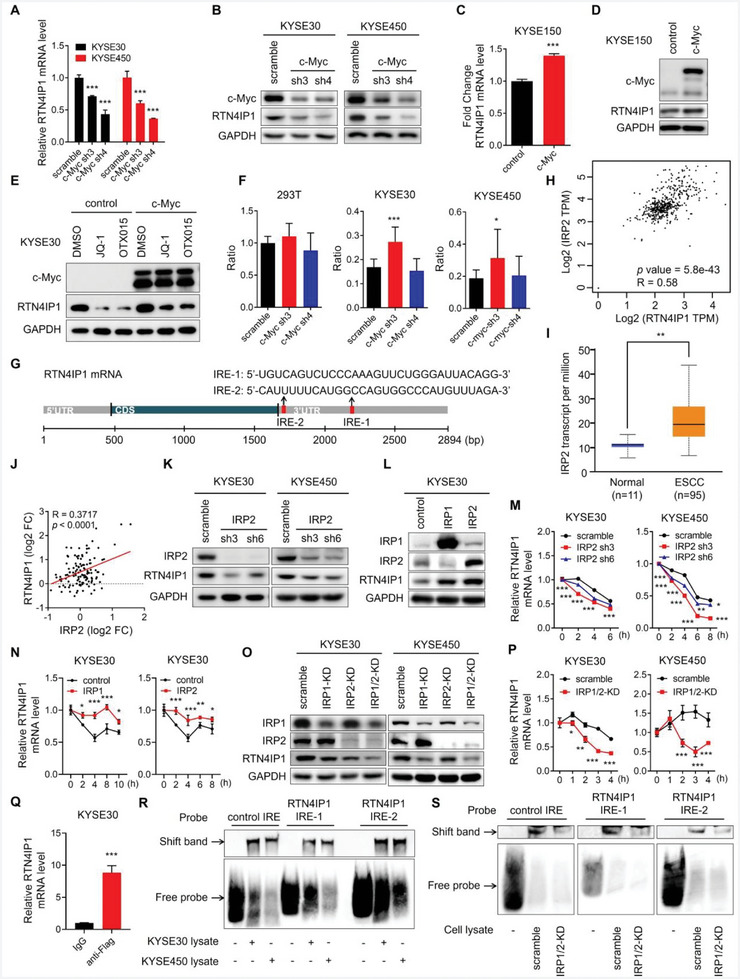
IRP‐IRE system modulates RTN4IP1 expression at mRNA stability level. A,B) KYSE30 and KYSE450 cells infected with lentivirus‐mediated shRNAs targeting c‐Myc or scramble were analyzed by RT‐PCR A) and Western blotting B). C,D) KYSE150 cell transfected with c‐Myc or control plasmids were analyzed by RT‐PCR C) and Western blotting D). E) KYSE30 cells transfected with c‐Myc or control plasmids for 48 h were treated with JQ‐1 (1 × 10^−6^ M) or OTX015 (1 × 10^−6^ M) for 24 h and then analyzed by Western blotting. F) Graphs showing the analysis of dual‐luciferase assay results of scramble or c‐Myc knockdown cells. Fluorescence value ratios of firefly luciferase to renilla luciferase were shown. Results from 3 independent biological replicates were statistically shown. G) Schematic of *RTN4IP1* mRNA with IRE in the 3′UTR. H) Graph showing correlation analysis of *RTN4IP1* and *IRP2* mRNA levels in ESCC by GEPIA. I) Graph showing mRNA expression pattern of IRP2 in ESCC, and normal esophageal tissues analyzed by UALCAN, *p = *0.0048. J) Graph showing correlation analysis of RTN4IP1 and IRP2 protein levels in ESCC proteomic study, *n = *124. K) KYSE30 and KYSE450 cells infected with lentivirus‐mediated shRNAs targeting IRP2 or scramble were analyzed by Western blotting. L) KYSE30 transfected with control, IRP1 or IRP2 plasmids were analyzed by Western blotting. M) KYSE30 and KYSE450 cells infected with lentivirus‐mediated shRNAs targeting IRP2 or scramble were treated with actinomycin D (1 µg mL^−1^) for indicated time and then analyzed by RT‐PCR. N) KYSE30 transfected with control, IRP1 or IRP2 plasmids were treated with actinomycin D (1 µg mL^−1^) for indicated time and then analyzed by RT‐PCR. O,P) KYSE30 and KYSE450 cells infected with lentivirus‐mediated shRNAs targeting IRP1, IRP2 or scramble were treated with actinomycin D (1 µg mL^−1^) for indicated time and then analyzed by Western blotting O) and RT‐PCR P). Q) RNA from KYSE30 cell transfected with IRP2‐Flag or control plasmids were immunoprecipitated by anti‐Flag antibody or IgG and then analyzed by RT‐PCR. R,S) Interaction between IRP1/2 and indicated IRE probes were determined by EMSA. Probes were incubated with indicated cytosolic lysate. In all statistical plots, data were expressed as the mean ± SD. *, *p <* 0.05, **, *p <* 0.01, ***, *p <* 0.001 by Student's *t*‐test (C, N, P, and Q) or Ordinary one‐way ANOVA (A, F, and M).

Given the essential role of RTN4IP1 in ESCC, we explored the mechanisms underlying RTN4IP1 upregulation. According to the cBio Cancer Genomics Portal (cBioPortal, http://cbioportal.org),^[^ ^23^ ^]^ *RTN4IP1* amplification occurs in a small percentage of esophageal cancer cases (Figure S3A, Supporting Information). As *RTN4IP1* mRNA level was significantly increased in ESCC tumor samples (Figure 1B,C), we speculated that RTN4IP1 might be upregulated at the transcriptional level. We demonstrated that RTN4IP1, a metabolism‐related protein, is regulated by BRD4. c‐Myc is a well‐known regulator of cell metabolism that functions downstream of BRD4. c‐Myc deregulation is a common feature of most human cancers.^[^ ^24^, ^25^ ^]^ Western blotting results showed that RTN4IP1 expression was consistent with that of c‐Myc in cells treated with BRD4 inhibitors (Figure S1H, Supporting Information). Further, we found a positive correlation between c‐Myc and RTN4IP1 in analyses using Gene Expression Profiling Interactive Analysis (GEPIA)^[^ ^26^ ^]^ and the Gene Expression Omnibus (GEO) database (Figure S3B, Supporting Information). Moreover, c‐Myc was upregulated in ESCC tissues compared with that in normal esophageal tissues (Figure S3C, Supporting Information). These findings prompted us to determine whether c‐Myc could regulate RTN4IP1 expression. Indeed, c‐Myc knockdown inhibited RTN4IP1 expression at both the mRNA and protein levels (**Figure** **3**A,B), whereas c‐Myc overexpression increased RTN4IP1 expression (Figure 3C,D). Furthermore, the ectopic expression of c‐Myc rescued RTN4IP1 downregulation following JQ‐1 and OTX015 treatment (Figure 3E). These data demonstrate that c‐Myc positively regulates RTN4IP1 expression. Next, we determined whether c‐Myc could directly regulate RTN4IP1 transcription. Unexpectedly, c‐Myc knockdown did not block the transcriptional activity of RTN4IP1 promoter in 293T, KYSE30, or KYSE450 cells (Figure 3F), suggesting that c‐Myc regulates RTN4IP1 expression at the post‐transcriptional level.

### 2.4 IRP–IRE System Modulates *RTN4IP1* mRNA Stability

Based on these results, we speculated that RTN4IP1 might be regulated at the mRNA‐stability level. Interestingly, we found two potential iron–responsive elements (IREs) in the 3′UTR of *RTN4IP1* mRNA predicted by SIREs Web Server 2.0^[^ ^27^ ^]^ (Figure 3G; Figure S3D, Supporting Information). Iron regulatory proteins (IRPs), including IRP1 and IRP2, are pivotal proteins that control cellular iron metabolism homeostasis. IRPs regulate the translation and stability of target mRNAs by binding to IREs.^[^ ^28^, ^29^ ^]^ IRP2 is a target of c‐Myc.^[^ ^30^ ^]^ Both IRP1 and IRP2 were significantly and positively correlated with RTN4IP1 expression (Figure 3H; Figure S3E, Supporting Information). IRP2, but not IRP1, was remarkably elevated in ESCC (Figure 3I; Figure S3F,G, Supporting Information) and significantly correlated with RTN4IP1 at the protein level (Figure 3J). However, elevated IRP1 predicted poor survival in patients with ESCC (Figure S3H, Supporting Information), suggesting a cancer‐promoting role of IRP1 in ESCC. Next, we determined the role of c‐Myc and IRPs in ESCC cell proliferation. Our results showed that overexpression of c‐Myc, IRP1, or IRP2 augmented cell viability, whereas knockdown of these proteins impaired cell proliferation and colony formation (Figure S3I–P, Supporting Information).

We further investigated the role of IRPs in RTN4IP1 expression. Our results showed that knockdown of IRP1 or IRP2 downregulated *RTN4IP1* mRNA and protein levels in KYSE30 and KYSE450 cells (Figure 3K; Figure S4A–C, Supporting Information), whereas overexpression of IRP1 or IRP2 elevated RTN4IP1 expression (Figure 3L; Figure S4D, Supporting Information). Next, we examined whether IRPs could influence *RTN4IP1* mRNA stability. Indeed, when cells were treated with actinomycin D for the global inhibition of mRNA synthesis, IRP1 or IRP2 knockdown significantly impaired *RTN4IP1* mRNA stability (Figure 3M; Figure S4E, Supporting Information), whereas IRP1 or IRP2 overexpression stabilized *RTN4IP1* mRNA (Figure 3N). Similar results were obtained in IRP1/2 double‐knockdown cells (Figure 3O,P; Figure S4F, Supporting Information). Collectively, these results demonstrate that IRP1/2 regulate *RTN4IP1* mRNA decay.

Next, we investigated whether IRP2 could directly bind to *RTN4IP1* mRNA. RNA immunoprecipitation assay confirmed a direct interaction between IRP2 and *RTN4IP1* mRNA (Figure 3Q). Furthermore, RTN4IP1–IRE probes exhibited shifted bands when incubated with KYSE30, KYSE450 and normal human liver lysates (Figure 3R; Figure S4G, Supporting Information), whereas impaired bandshifts were observed when IRP1/2 was knocked down (Figure 3S), implying a direct interaction between RTN4IP1 IREs and IRP1/2 proteins. Collectively, our results confirm that the IRP–IRE system modulates RTN4IP1 expression via the control of mRNA stability in ESCC cells.

### 2.5 c‐Myc Regulates *RTN4IP1* mRNA Stability via the IRP–IRE System

**Figure 4:**
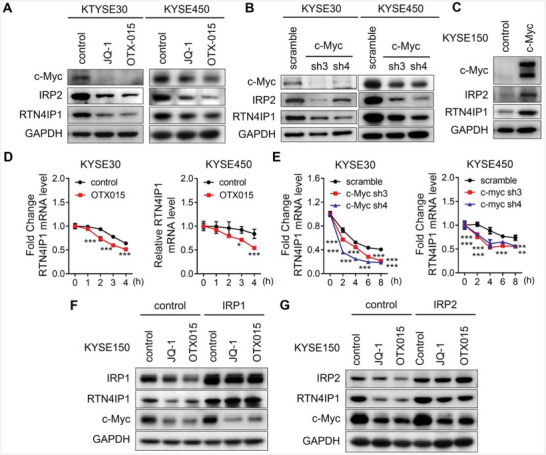
c‐Myc modulates *RTN4IP1* mRNA stability via IRP2‐IRE system. A) KYSE30 and KYSE450 cells treated with JQ‐1 (1 × 10^−6^ M) or OTX015 (1 × 10^−6^ M) for 24 h were analyzed by Western blotting. B) KYSE30 and KYSE450 cells infected with lentivirus‐mediated shRNAs targeting c‐Myc or scramble were analyzed by Western blotting. C) KYSE150 cell transfected with c‐Myc overexpressing or control plasmids were analyzed by Western blotting. D) KYSE30 and KYSE450 cells treated with OTX015 (1 × 10^−6^ M) for 16 h and actinomycin D (1 µg mL^−1^) for indicated time were analyzed by RT‐PCR. E) KYSE30 and KYSE450 cells infected with lentivirus‐mediated shRNAs targeting c‐Myc or scramble were treated with actinomycin D (1 µg mL^−1^) for indicated time and then analyzed by RT‐PCR. F,G) KYSE150 cells transfected with IRP1, IRP2 or control plasmids for 48 h were treated with JQ‐1 (1 × 10^−6^ M) or OTX015 (1 × 10^−6^ M) for 24 h, then analyzed by Western blotting. In all statistical plots, data were expressed as the mean ± SD. *, *p <* 0.05, **, *p <* 0.01, ***, *p <* 0.001 by Student's *t*‐test (D) or Ordinary one‐way ANOVA (E).

As previously reported, c‐Myc stimulates IRP2 expression.^[^ ^30^ ^]^ Consistent with this, c‐Myc downregulation resulting from JQ‐1, OTX015 treatment, or shRNA‐mediated specific knockdown suppressed IRP2 expression in KYSE30 and KYSE450 cells (**Figure** **4**A,B), whereas elevated IRP2 expression was observed in response to c‐Myc overexpression (Figure 4C). Hence, we investigated whether c‐Myc could regulate RTN4IP1 expression via the IRP2–IRE–mediated inhibition of mRNA decay. We found that *RTN4IP1* mRNA stability significantly decreased upon OTX015 administration (Figure 4D). Similarly, increased mRNA decay was observed in c‐Myc–knockdown cells (Figure 4E). These results indicate that c‐Myc can manipulate *RTN4IP1* mRNA stability. Next, to determine whether the IRP–IRE system serves as a downstream effector of c‐Myc, we conducted forced IRP1/2 overexpression in JQ‐1‐ and OTX015‐treated cells. As expected, IRP1/2 overexpression blocked RTN4IP1 downregulation induced by JQ‐1 or OTX015 treatment (Figure 4F,G). Taken together, these data suggest that c‐Myc regulates *RTN4IP1* mRNA stability via the IRP2–IRE system in ESCC cells.

Further, we analyzed the correlations among *RTN4IP1*, *c‐Myc*, and *IRP2*, and assessed the expression patterns of these genes in thyroid and breast cancers. We found that all three genes were downregulated, and *IRP2* showed a significant positive correlation with *RTN4IP1* in thyroid cancer, whereas only *RTN4IP1* expression was elevated, and no significant correlation was observed between these three genes in breast cancer (data not shown). These preliminary analyses suggest that the c‐Myc–IRP2 axis contributes to RTN4IP1 elevation, specifically in ESCC (and thyroid cancer), and other mechanisms may be responsible for RTN4IP upregulation in breast cancer, such as promoter methylation and gene amplification.

### 2.6 RTN4IP1 Deficiency Leads to Amino Acid Starvation and Downregulation of Amino Acid Transporters in ESCC

**Figure 5:**
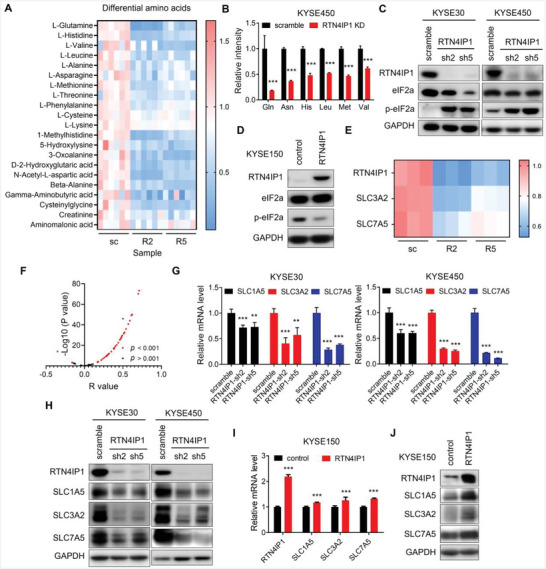
RTN4IP1 depletion results in amino acid starvation via downregulation of SLC1A5, SLC3A2, and SLC7A5. A) Heatmap showing differential amino acids in RTN4IP1‐knockdown (R2, R5) or scramble (sc) KYSE450 cells in metabolomics data. B) Amino acid levels in scramble and RTN4IP1‐KD KYSE450 cells were analyzed by LC‐MS/MS. C) KYSE30 and KYSE450 cells infected with lentivirus‐mediated shRNAs targeting RTN4IP1 or scramble were analyzed by Western blotting. D) KYSE150 cells transfected with RTN4IP1 or control plasmids were analyzed by Western blotting. E) Heatmap showing expression of indicated proteins in scramble (sc) and RTN4IP1‐knockdown (R2, R5) KYSE450 cells in the proteomics data. F) Graph showing correlation analysis between RTN4IP1 and 60 amino acid transporters based on GEPIA analysis. G,H) KYSE30 and KYSE450 cells infected with lentivirus‐mediated shRNAs targeting RTN4IP1 or scramble were analyzed by RT‐PCR G) and Western blotting H). I,J) KYSE150 cells transfected with control or RTN4IP1 plasmids were analyzed by RT‐PCR I) and Western blotting J). In all statistical plots, data were expressed as the mean ± SD. **, *p <* 0.01, ***, *p <* 0.001 by Student's *t*‐test (B and I) or Ordinary one‐way ANOVA (G).

Recently, RTN4IP1 was reported to be an oxidoreductase that may participate in CoQ biosynthesis.^[^ ^14^ ^]^ CoQ plays an essential role as an electron shuttle in the respiratory chain and a cofactor of several important dehydrogenases, and is a crucial antioxidant to protect cells from oxidative stress and ferroptosis.^[^ ^31^, ^32^, ^33^ ^]^ Besides, dihydroorotate dehydrogenase employs CoQ in *de novo* pyrimidine synthesis.^[^ ^34^, ^35^ ^]^ Therefore, we speculated that RTN4IP1 depletion might result in oxidative stress and CoQ or nucleotide shortage, thus impairing cell viability. However, the supplementation of N‐acetyl cysteine (NAC), CoQ10 or Inosine 5′‐monophosphate (IMP) and Uridine 5′‐monophosphate (UMP), could not efficiently rescue cell growth inhibition upon RTN4IP1 silencing (Figure S5A,B, Supporting Information), implying the involvement of RTN4IP1 in other cellular process essential to cell survival and growth. Given that RTN4IP1 may function as an oxidoreductase, we conducted metabolomic and proteomic analyses to explore the biological effects caused by RTN4IP1 depletion. Unexpectedly, our metabolomic data showed a clear reduction in amino acids in RTN4IP1‐knockdown cells (**Figure** **5A**; Figure S5C, Supporting Information), which was verified using liquid chromatography tandem mass spectrometry (LC‐MS/MS) (Figure 5B). The decreased amino acid levels and apoptotic phenotypes caused by RTN4IP1 knockdown led us to investigate the integrated stress response (ISR) pathway, which can be activated by various intrinsic and extrinsic stresses (such as amino acid depletion) and functions at the crossroads of cell survival and apoptosis. Amino acid starvation can be sensed by general control nonderepressible 2 (GCN2) kinase and that subsequently induces phosphorylation of eukaryotic translation initiation factor 2A (eIF2α), thus inhibiting global protein synthesis and triggering the translation of specific mRNAs to help with cell survival and recovery; if cells fail to redress homeostasis, ISR shifts to promote apoptosis.^[^ ^36^, ^37^ ^]^ To determine whether RTN4IP1 silencing results in ISR activation, we examined phosphorylation of eIF2α. Indeed, we observed increased eIF2α phosphorylation in response to RTN4IP1 depletion (Figure 5C) and downregulated eIF2α phosphorylation upon RTN4IP1 overexpression (Figure 5D). Taken together, we conclude that RTN4IP1 knockdown results in amino acid starvation and activation of ISR pathway in ESCC cells.

To elucidate how RTN4IP1 depletion reduces the amino acid pool, we analyzed our proteomic data and found that two amino acid transporters, solute carrier family 3 member 2 (SLC3A2) and solute carrier family 7 member 5 (SLC7A5), were significantly decreased in RTN4IP1‐knockdown cells (Figure 5E). To further elucidate the role of RTN4IP1 in amino acid transport, we analyzed the correlation between RTN4IP1 and known amino acid transporters. Interestingly, 28 transporters exhibited a positive correlation with RTN4IP1 (R ≥ 0.3, *p <* 0.001) (Figure 5F; Table S1, Supporting Information), indicating that RTN4IP1 might be closely related with amino acid transport. Next, we determined the mRNA levels of these amino acid transporters in RTN4IP1‐deficient cells. In addition to SLC3A2 and SLC7A5, 12 other amino acid transporters showed reduced mRNA levels upon RTN4IP1 knockdown in KYSE450 cell (Figure 5G; Figure S5D, Supporting Information). To screen for the key amino acid transporters downstream of RTN4IP1 in ESCC, we examined the expression patterns of 14 downregulated transporters and found that nine of them were significantly elevated in ESCC compared with normal esophageal tissues (Figure S5E, Supporting Information). Solute carrier family 1 member 5 (SLC1A5) is a major transporter of glutamine implicated in cancers.^[^ ^38^, ^39^, ^40^, ^41^ ^]^ Consistent with this, we observed a marked decrease in glutamine level in RTN4IP1‐deficient cell (Figure 5A,B). SLC3A2 and SLC7A11 are subunits of the system x_c_^−^ which import extracellular cystine. Subsequently, cystine is converted to cysteine and utilized to synthesize glutathione (GSH), which is essential for redox maintenance.^[^ ^42^ ^]^ However, we did not observe a notable decline in cystine or cysteine levels in RTN4IP1‐knockdown cells (Figure 5A; Figure S5F, Supporting Information). SLC25A22 is a mitochondrial glutamate transporter that facilitates the transport of glutamate to the mitochondrial matrix.^[^ ^43^ ^]^ We focused on the transporters localized on the cell membrane, which are thought to be the main contributors to the cellular amino acid pool. Based on the substrate specificity, expression patterns in ESCC and metabolomic data from our study, we selected SLC1A5, SLC3A2, and SLC7A5 for further investigation.

We observed profoundly reduced SLC3A2 and SLC7A5 levels upon RTN4IP1 knockdown, as well as the downregulated SLC1A5 (Figure 5G,H). In contrast, RTN4IP1 overexpression led to the upregulation of SLC1A5, SLC3A2, and SLC7A5 (Figure 5I,J).

### 2.7 SLC1A5, SLC3A2, and SLC7A5 are Significantly Upregulated in ESCC and Contribute to Tumor Growth

**Figure 6:**
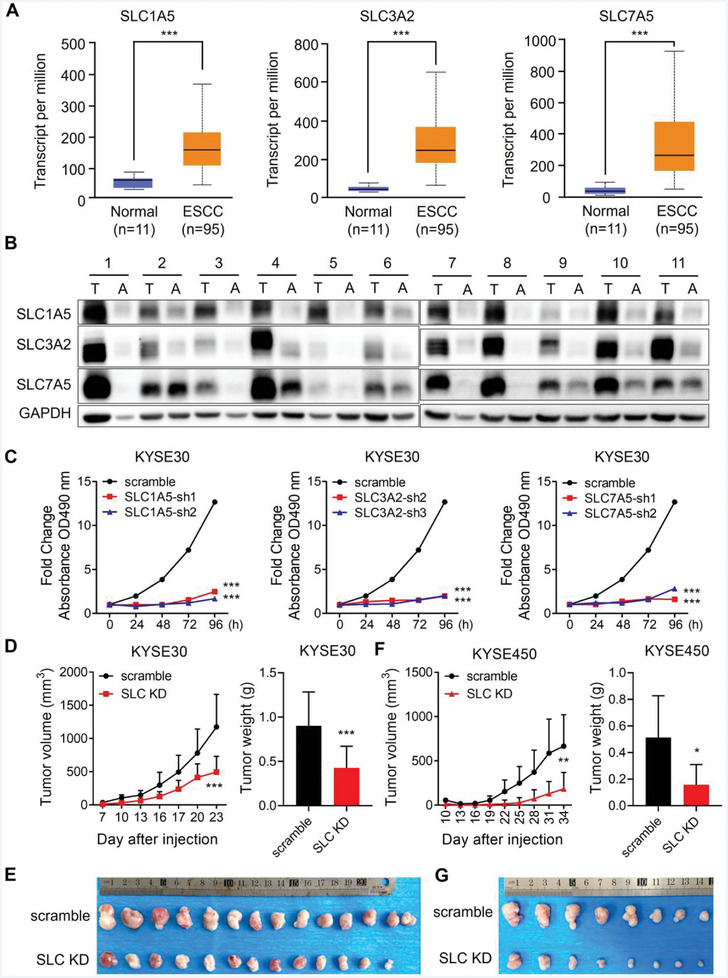
SLC1A5, SLC3A2, and SLC7A5 are upregulated in ESCC and essential for cell proliferation. A) Graphs showing mRNA expression pattern of SLC1A5, SLC3A2, and SLC7A5 in ESCC and normal esophageal tissues analyzed by UALCAN, *p = *2.43e‐13, *p = *3.35e‐10, and *p = *1.89e‐05, respectively. B) Western blotting analysis of indicated protein levels in paired ESCC and adjacent tissues. A, adjacent tissue; T, tumor tissue, *n = *11 pairs. C) KYSE30 infected with lentivirus‐mediated shRNAs targeting SLC1A5, SLC3A2, SLC7A5 or scramble were analyzed by MTT assay. D‐G) Growth curves and weight data of xenograft tumors derived from indicted KYSE30 and KYSE450 cells D,F). Volumes of xenograft tumors were measured at indicated time and showed statistically. Images showed xenograft tumors E,G). In all statistical plots, data were expressed as the mean ± SD. *, *p <* 0.05, **, *p <* 0.01, ***, *p <* 0.001 by Student's *t*‐test (D and F) or Ordinary one‐way ANOVA (C).

We investigated the expression of SLC1A5, SLC3A2, and SLC7A5 in ESCC. As shown by TCGA data, the three transporters were upregulated in ESCC (**Figure** **6**A). Analysis of proteomic data from clinical ESCC specimens also revealed significantly elevated expression levels of SLC1A5, SLC3A2, and SLC7A5 in ESCC (Figure S6A, Supporting Information). Moreover, remarkable upregulation of SLC1A5, SLC3A2, and SLC7A5 were revealed by Western blotting in paired ESCC tumor and normal tissues (Figure 6B). Next, we investigated the effects of SLC1A5, SLC3A2, and SLC7A5 on cell proliferation and observed that cell proliferation and colony formation abilities were profoundly hampered when the amino acid transporters were individually knocked down (Figure 6C; Figure S6B–E, Supporting Information). Furthermore, we evaluated the effect of the simultaneous knockdown of SLC1A5, SLC3A2, and SLC7A5 on tumor growth in a cell‐derived xenograft (CDX) mouse model. The results showed that SLC1A5/SLC3A2/SLC7A5 knockdown diminished tumor growth in vivo (Figure 6D–G). Based on these results, we conclude that SLC1A5, SLC3A2, and SLC7A5 contribute to ESCC carcinogenesis.

### 2.8 RTN4IP1 Manipulates Cell Proliferation via SLC1A5/SLC3A2/SLC7A5

**Figure 7:**
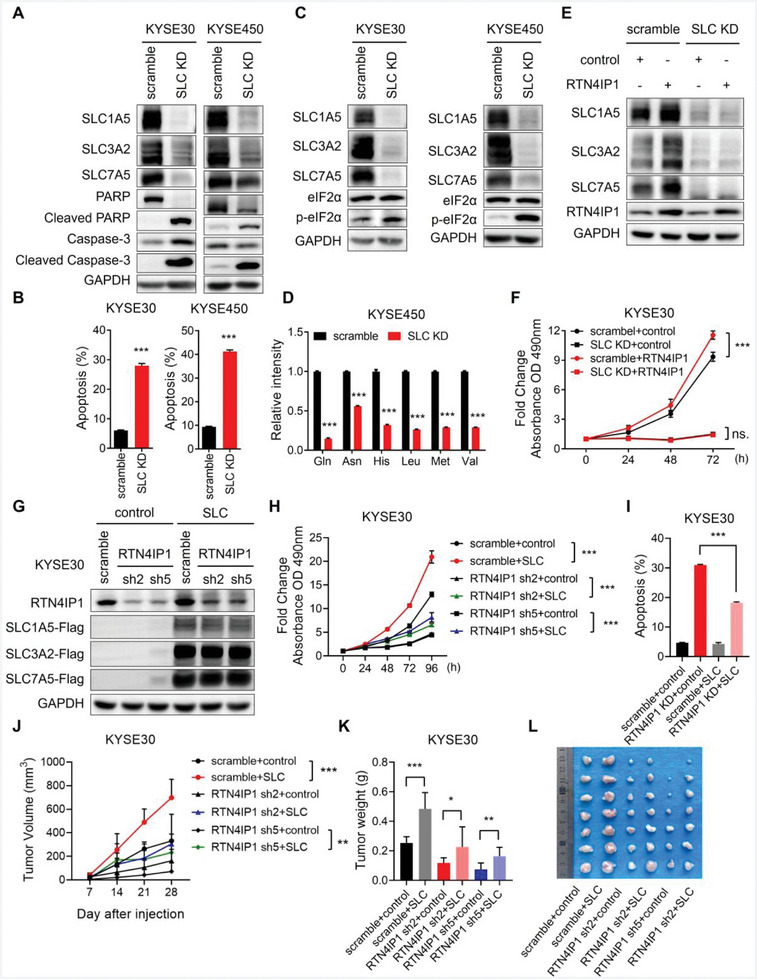
RTN4IP1 manipulates cell proliferation via SLC1A5/SLC3A2/SLC7A5. A–D) KYSE30 and KYSE450 cells co‐infected with lentivirus‐mediated shRNAs targeting SLC1A5/SLC3A2/SLC7A5 or scramble were analyzed by A,C) Western blotting, B) Flow cytometry and D) LC‐MS/MS. E,F) KYSE30 cells co‐infected with lentivirus‐mediated shRNAs targeting SLC1A5/SLC3A2/SLC7A5 or scramble were transfected with control or RTN4IP1 overexpressing plasmids and then analyzed by E) Western blotting and F) MTT assay. G‐I) KYSE30 cells infected with lentivirus‐mediated shRNAs targeting RTN4IP1 or scramble were transfected with lentivirus‐mediated overexpression of control or SLC1A5/SLC3A2/SLC7A5 and then analyzed by G) Western blotting, H) MTT and I) Flow cytometry. J) Cells from G were injected into nude mice to generate tumors. Volumes of xenograft tumors were measured at indicated time and showed statistically in tumor growth curves. K) Graph showed analysis of the weight of xenograft tumors at endpoint after subcutaneous cell injection. L) Image showed xenograft tumors of indicated groups. In all statistical plots, data were expressed as the mean ± SD. ***, *p <* 0.001 by Student's *t*‐test (B, D, J, and K) or Ordinary one‐way ANOVA (F, H and I).

Based on the correlation between RTN4IP1 and amino acid transporters, we investigated whether SLC1A5/SLC3A2/SLC7A5 knockdown could phenocopy RTN4IP1 depletion. We noticed that SLC1A5/SLC3A2/SLC7A5 knockdown induced apoptosis and cell cycle arrest in ESCC cell lines (**Figure** **7**A,B; Figure S7A,B, Supporting Information). Nevertheless, augmented eIF2α phosphorylation was observed upon SLC1A5/SLC3A2/SLC7A5 depletion (Figure 7C). As shown by the LC‐MS/MS result, SLC1A5/SLC3A2/SLC7A5 knockdown significantly reduced the amino acid pool (Figure 7D).

To further confirm the relationship between RTN4IP1 and amino acid uptake, we depleted SLC1A5/SLC3A2/SLC7A5 in RTN4IP1‐overexpressing cells (Figure 7E). The MTT assay results showed that cell proliferation augmentation resulting from overexpression of RTN4IP1 was blocked in the absence of SLC1A5/SLC3A2/SLC7A5 (Figure 7F). In contrast, supplementation of SLC1A5/SLC3A2/SLC7A5 in RTN4IP1‐deficient cell significantly rescued cell proliferation (Figure 7G,H). Moreover, SLC1A5/SLC3A2/SLC7A5 overexpression repressed apoptosis and restored DNA synthesis in RTN4IP1‐knockdown cell (Figure 7I; Figure S7C, Supporting Information). The results from the CDX mouse model also demonstrated that SLC1A5/SLC3A2/SLC7A5 supplementation promoted the growth of tumors derived from scramble or RTN4IP1 knockdown cells (Figure 7J–L). Collectively, these data reveal that SLC1A5, SLC3A2, and SLC7A5 are essential downstream effectors of RTN4IP1, highlighting the role of RTN4IP1 in amino acid uptake in ESCC.

SLC1A5 and SLC7A5 are targets of c‐Myc.^[^ ^44^, ^45^ ^]^ c‐Myc knockdown led to the reduced expression of SLC1A5/SLC3A2/SLC7A5 and ISR activation in ESCC cells (Figure S7D, Supporting Information). To determine whether RTN4IP1 can regulate SLC1A5/SLC3A2/SLC7A5 via c‐Myc, we measured c‐Myc expression following RTN4IP1 knockdown. The results showed that RTN4IP1 knockdown did not reduce c‐Myc expression (Figure S7E, Supporting Information), suggesting that RTN4IP1 may regulate SLC1A5/SLC3A2/SLC7A5 in a c‐Myc‐independent manner. We also found that c‐Myc overexpression elevated SLC1A5/SLC3A2/SLC7A5 expression in RTN4IP1‐deficient cells, and promoted cell proliferation as well as tumor growth upon RTN4IP1 knockdown, demonstrating the essential role of SLC1A5/SLC3A2/SLC7A5 in RTN4IP1‐mediated cell proliferation (Figure S7F–J, Supporting Information).

In addition, we determined the expression of these three amino acid transporters following IRP1/2 knockdown. Reduced levels of SLC1A5, SLC3A2, and SLC7A5, as well as elevated eIF2α phosphorylation were observed in IRP1/2‐knockdown cells (Figure S7K, Supporting Information), which was in consistent with the effects observed with RTN4IP1 deficiency.

### 2.9 RTN4IP1 Deficiency Suppresses ESCC Progression In Vivo

**Figure 8:**
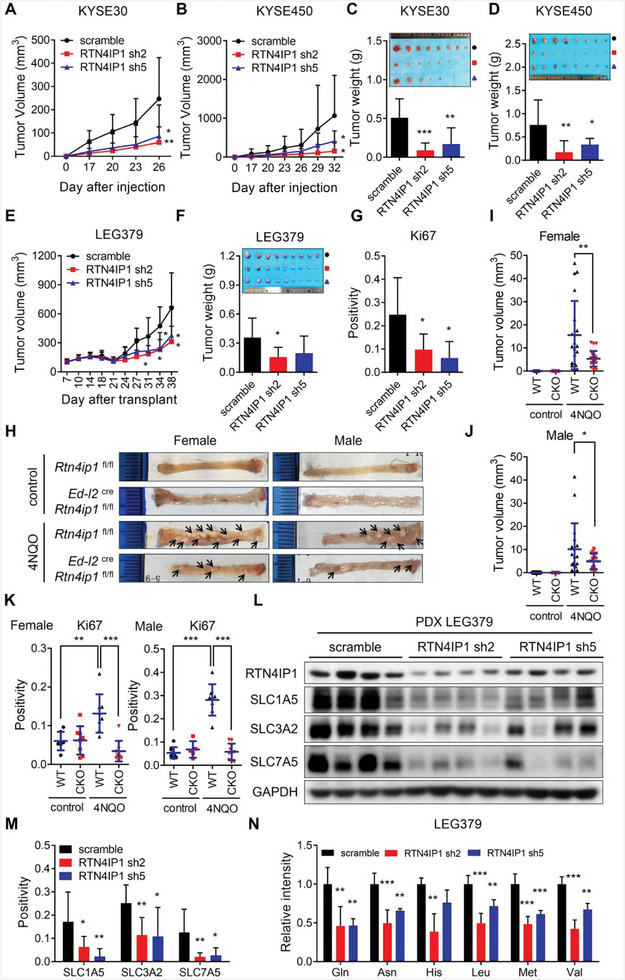
RTN4IP1 deficiency suppresses ESCC progression in vivo. A) Tumor growth curves of tumors derived from indicted KYSE30 and B) KYSE450 cells. Volumes of xenograft tumors were measured at indicated time and showed statistically. Graphs showing analysis of the weight of xenograft tumors depicted in panel A and C at endpoint after subcutaneous cell injection. Images on the top showed xenograft tumors derived from indicted C) KYSE30 and D) KYSE450 cells. E–G) Tumors derived from LEG379 PDX model were transplanted and then injected with lentivirus targeting RTN4IP1 and scramble. Tumor volumes were measured and at indicated time. E) Growth curve and F) the weight of PDX tumors at endpoint were statistically showed. Image on the top of panel F showed the PDX tumors. G) Graph showing the analysis of IHC staining of Ki67 from PDX tumors. H) Representative images of esophagi from indicated *Rtn4ip1* ^fl/fl^ (WT) and *Ed‐l2*cre *Rtn4ip1* ^fl/fl^ (CKO) mice. Arrows showed tumors on the luminal surface of esophagi. Quantification of I‐J) tumor volumes and K) Ki67 staining of esophagi were shown. L) Western blotting analysis of tumors derived from LEG379 PDX model subjected to lentivirus injection. M) Graph showing the analysis of IHC staining of indicated proteins in LEG379 PDX tumors. N) Tumors from LEG379 PDX model subjected to lentivirus injection were analyzed by LC‐MS/MS. In all statistical plots, data were expressed as the mean ± SD. *, *p <* 0.05, **, *p <* 0.01, ***, *p <* 0.001 by Ordinary one‐way ANOVA.

To determine the effects of RTN4IP1 on ESCC development, we delivered RTN4IP1‐knockdown KYSE30 and KYSE450 cells into female athymic nude mice via subcutaneous injection. Overall, tumors derived from RTN4IP1‐knockdown cells grew much slower and exhibited decreased size and weight than the control groups (**Figure** **8**A–D). Moreover, we generated and delivered lentivirus‐bearing shRNAs targeting RTN4IP1 or scramble into ESCC patient‐derived xenograft (PDX) to achieve in vivo knockdown of RNT4IP1. Our data showed that effective RTN4IP1 depletion in PDX tumors resulted in significant inhibition of tumor growth (Figure 8E,F; Figure S8A,B, Supporting Information), along with a significant decrease of Ki67‐positive malignant cells in RTN4IP1‐knockdown tumors (Figure 8G; Figure S8C, Supporting Information).

To better illustrate the effect of RTN4IP1 on ESCC development, we generated esophageal‐specific *Rtn4ip1*‐knockout (CKO) mice by crossing *Rtn4ip1* ^fl/fl^ mice with *Ed‐l2* ^cre^ mice and then induced ESCC using the carcinogen 4‐nitroquinoline 1‐oxide (4‐NQO). Consistently, *Ed‐l2* ^cre^ *Rtn4ip1* ^fl/fl^ mice developed fewer and smaller tumors upon 4‐NQO treatment than their wild‐type littermate *Rtn4ip1* ^fl/fl^ mice (Figure 8H–J; Figure S8D, Supporting Information). The thicker and shorter esophagi resulting from 4‐NQO treatment were alleviated in *Rtn4ip1* CKO mice (Figure S8E, Supporting Information). RTN4IP1 deletion also weakened Ki67 staining in 4‐NQO‐induced tumors (Figure 8K; Figure S8F, Supporting Information). Based on these results, we conclude that RTN4IP1 deficiency inhibits ESCC development in vivo.

To determine whether RTN4IP1 can regulate SLC1A5, SLC3A2, and SLC7A5 in vivo, we examined the expression of these transporters in tumors from the LEG379 PDX model. Western blotting results showed that RTN4IP1 silencing caused by lentivirus injection resulted in the downregulation of SLC1A5, SLC3A2, and SLC7A5 (Figure 8L). The immunohistochemical results were consistent with those of Western blotting (Figure 8M; Figure S8C, Supporting Information). Moreover, diminished levels of amino acids in RTN4IP1‐knockdown tumors were detected (Figure 8N). In summary, our findings demonstrate the regulatory role of RTN4IP1 in amino acid uptake in vivo.

## 3 Discussion

Accumulating evidence has confirmed the vital role of metabolic reprogramming in multiple stages of tumorigenesis, providing new opportunities to improve cancer therapy.^[^ ^46^, ^47^, ^48^ ^]^ However, metabolic alterations in ESCC remain unclear. Recent studies have revealed the emerging importance of RTN4IP1 in cancer; however, its role is not yet completely understood. Here, we present evidence of *RTN4IP1* as a cancer‐promoting gene that is profoundly upregulated and essential for cell proliferation in ESCC. We further demonstrate that c‐Myc–IRP2–IRE axis is responsible for the upregulation of RTN4IP1, and identify SLC1A5, SLC3A2, and SLC7A5, three amino acid transporters implicated in cancer, as essential downstream effectors of RTN4IP1 in ESCC. Overall, our findings indicate that elevated RTN4IP1 contributes to ESCC development by augmenting amino acid uptake (Figure S9, Supporting Information).

The deregulated expression of cancer‐related genes plays an important role in cancer pathogenesis. Known as key regulators of iron homeostasis, the expression of IRP1/2, especially IRP2, is altered in several types of human cancer.^[^ ^49^, ^50^, ^51^ ^]^ However, the mechanisms by which IRP1/2 contribute to tumorigenesis remains unclear. In this study, we identified RTN4IP1 as a direct target of IRP1/2. Our results revealed a mechanistic connection between IRP1/2 and amino acid uptake. IRP1 is a bifunctional protein that serves as either a high‐affinity IRE‐binding protein (without the [4Fe–4S] cluster) or a cytosolic aconitase (with the [4Fe–4S] cluster). Unlike IRP1, IRP2 lacks a [4Fe–4S] cluster and aconitase activity, and functions as an RNA‐binding protein. IRP2 is primarily regulated by iron‐mediated degradation, and IRP1 responds to iron‐dependent and iron‐independent signals that control its function through multiple mechanisms to maintain suitable IRE‐binding activity for cellular iron homeostasis.^[^ ^28^, ^29^ ^]^ Notably, although IRP1 is not differentially expressed between tumor and normal tissues in ESCC, it is still able to promote cell proliferation, and upregulation of IRP1 predicts unfavorable outcomes in patients with ESCC, implying a possible oncogenic role of IRP1. In this study, we revealed that IRP1 could directly regulate *RTN4IP1* mRNA stability in ESCC. We hypothesized the involvement of both IRP1 and IRP2 in regulating *RTN4IP1* mRNA stability in ESCC cells. Even though the expression of IRP1 was not significantly elevated in ESCC, the IRE‐binding activity of IRP1 might be upregulated, which may contribute to the increased expression of RTN4IP1.

Multiple steps are involved in gene expression, including gene alterations, epigenetic regulation, transcription factors, mRNA decay, and translational control, as well as protein stability. The upstream regulatory mechanisms of RTN4IP1 may be complex and vary with tumor type. We found that RTN4IP1 is regulated by different mechanisms in ESCC and breast cancer, and that the mechanism may be common for ESCC and thyroid cancer.

We revealed that RTN4IP1 is closely related to amino acid transport. SLC1A5, SLC3A2, and SLC7A5 are well‐documented amino acid transporters that mediate the cross‐membrane flux of large neutral amino acids which are reported to be upregulated and act as contributors in different types of cancer.^[^ ^38^, ^52^, ^53^, ^54^ ^]^ To date, ≈60 members of the solute carrier family have been reported to be involved in cross‐membrane amino acid transport in mammalian cells.^[^ ^55^ ^]^ Interestingly, nearly half of the known amino acid transporters positively correlated with RTN4IP1 in ESCA, of which 14 were downregulated upon RTN4IP1 deficiency. We demonstrated that RTN4IP1 could positively regulate the expression of SLC1A5, SLC3A2, and SLC7A5, and RTN4IP1 silencing could induce amino acid starvation. Additionally, SLC1A5/SLC3A2/SLC7A5 knockdown produced the same effects as RTN4IP1 depletion. Based on our results, RTN4IP1 deficiency or SLC1A5/SLC3A2/SLC7A5 knockdown results in cell cycle arrest and apoptosis. Adequate supply of amino acid is essential for nucleotide availability for cell cycle progression.^[^ ^56^, ^57^, ^58^ ^]^ Accordingly, our metabolomics data indicated that RTN4IP1 knockdown reduced nucleoside levels (data not shown). We further demonstrated that SLC1A5/SLC3A2/SLC7A5 overexpression could restore DNA synthesis and cell viability in RTN4IP1 knockdown cells, highlighting the role of amino acid uptake as a linchpin in RTN4IP1‐mediated cell proliferation. These findings demonstrated that RTN4IP1 promotes tumor growth by increasing amino acid uptake in ESCC.

SLC1A5, SLC3A2, and SLC7A5 are upregulated in various cancer types,^[^ ^38^, ^39^, ^40^, ^41^, ^52^, ^53^, ^54^ ^]^ which underpins the increased demand for bulk protein synthesis owing to the enhanced proliferation of cancer cells. However, the detailed mechanism by which RTN4IP1 regulates the expression of SLC1A5, SLC3A2, and SLC7A5 requires further investigation. In addition, SLC1A5/SLC3A2/SLC7A5 partially restored cell viability in RTN4IP1‐deficient cell, therefore, we cannot exclude the possibility that other processes may be involved in RTN4IP1‐mediated proliferation control.

Amino acid transport plays a crucial role in regulating immune responses in the tumor microenvironment and is regarded as an attractive target for cancer immunity.^[^ ^59^, ^60^ ^]^ The possible role of RTN4IP1 in the tumor microenvironment, not only in cancer cells, deserves in‐depth exploration. More recently, it has been reported that SLC7A5‐mediated amino acid transport is crucial for neuronal excitability and survival,^[^ ^61^ ^]^ in line with a previous study showing that SLC7A5 mutations cause autism via the disruption of normal levels of brain branch chain amino acids (BCAAs) in humans,^[^ ^62^ ^]^ suggesting a key role of SLC7A5 in neuronal viability and function. On the other hand, *RTN4IP1* mutations are associated with recessive optic neuropathy and additional neurological symptoms in humans. It will be interesting to determine whether RTN4IP1 is involved in amino acid transport by regulating the expression of amino acid transporters in neurons.

In addition to SLC1A5, SLC3A2, and SLC7A5, we found 11 additional amino acid transporters downregulated upon RTN4IP1 knockdown. Interestingly, SLC16A10, which mediates the cross‐membrane transport of thyroid hormones, was downregulated upon RTN4IP1 knockdown. RTN4IP1 is a tumor suppressor in thyroid cancer;^[^ ^15^ ^]^ however, the underlying mechanism remains unknown. Our findings shed light on the role of RTN4IP1 in thyroid cancer. Besides amino acid transporters that reside on the cell membrane, we also found that the mitochondrial transporters SLC25A13 and SLC25A22 were reduced in RTN4IP1 knockdown cells, indicating a possible impairment of cytosol–mitochondria amino acid transport upon RTN4IP1 deficiency. Further studies on these amino acid transporters will help elucidate the role of RTN4IP1 in cellular amino acid transport.

In conclusion, our study revealed that ESCC cells achieve elevated RTN4IP1 expression through mRNA stabilization facilitated by the c‐Myc–IRP2–IRE axis. RTN4IP1 may contribute to ESCC carcinogenesis via regulating amino acid influx mediated by amino acid transporters. Further investigations into RTN4IP1 will be instrumental in unveiling its detailed biochemical function and developing specific inhibitors of RTN4IP1, which may be promising options to overcome the limitations of ESCC therapy.

## 4 Experimental Section

### Cell Culture

All cell lines were gained from the Type Culture Collection of the Chinese Academy of Sciences (Shanghai, China) and cultured at 37 °C in 5% CO_2_. ESCC cell lines KYSE30, KYSE150, KYSE450 were maintained in RPMI 1640 medium (Biological Industries) containing 10% FBS (Biological Industries) and 1% penicillin/streptomycin (Solarbio). 293T cell was maintained in high glucose DMEM medium (Biological Industries) supplemented with 10% FBS, 1% penicillin/streptomycin. Cell lines were authenticated by short tandem repeat (STR) fingerprinting.

### Reagents and Antibodies

JQ‐1 and OTX015 were purchased from Rechem, Shanghai. Actinomycin D and 4‐NQO were purchased from Sigma. The following primary antibodies were used for immunoblot, anti‐RTN4IP1 (ORIGENE, TA502544, 1:1000), anti‐PARP (Cell signaling technology, #9542, 1:1000), anti‐cleaved PARP (Cell signaling technology, #5625, 1:1000), anti‐Caspase‐3 (Cell signaling technology, #9662, 1:1000), anti‐Cleaved Caspase‐3 (Cell signaling technology, #9664, 1:1000), anti‐c‐Myc (Abcam, ab32072, 1:1000), anti‐IRP1 (Abcam, ab236773, 1:1000), anti‐IRP2 (Cell signaling technology, #37135, 1:1000), anti‐SLC1A5 (Cell signaling technology, #8057, 1:1000), anti‐SLC3A2 (Santa Cruz Biotechnology, sc‐376815, 1:100), anti‐SLC7A5 (Cell signaling technology, #5347, 1:1000), anti‐eIF2α (Cell signaling technology, #9722, 1:1000), anti‐p‐eIF2α (Cell signaling technology, #9721, 1:1000), and anti‐GAPDH (Proteintech, #HRP‐60004, 1:3000). The following antibodies were used for IHC, anti‐RTN4IP1 (ORIGENE, TA502544, 1:50), anti‐Ki67 (Abcam, ab16667, 1:50), anti‐SLC1A5 (Cell signaling technology, #8057, 1:1000), anti‐SLC3A2 (Santa Cruz Biotechnology, sc‐376815, 1:100), anti‐SLC7A5 (Cell signaling technology, #5347, 1:100).

### Plasmids and Lentivirus

Human RTN4IP1, c‐Myc, IRP1 and IRP2 were cloned into indicated vectors, details were shown in Table S2 (Supporting Information). SLC1A5, SLC3A2, and SLC7A5 in pCDH‐CMV‐MCS‐EF1‐Puro‐3×Flag vectors were purchased from Youbio (Changsha, Hunan, China). shRNA plasmids were constructed with pLKO.1 vector using primers shown in Table S3 (Supporting Information). Lentiviruses used for stable knockdown of indicated genes were packaged by transfection of psPAX2, pMD2.G and pLKO.1‐based shRNA plasmids into 293T cell using jetPRIME (Polyplus‐transfection, #114‐15). Medium was changed after 5‐h incubation. Lentivirus supernatant was collected after 48‐h transfection. Polybrene (8 µg mL^−1^, Solarbio, H8761) was used for lentivirus infection. Cells were subjected to puromycin (2 µg mL^−1^ Solarbio, P8230) for 48‐h selection after 24‐h infection.

### Western Blotting

Cells were collected at the end of treatment and protein extracts were prepared by using RIPA lysis buffer (5 × 10^−2^ M Tris (pH 7.4), 1.5 × 10^−1^ M NaCl, 1% NP‐40, 0.5% sodium deoxycholate, 1× 10^−3^ M EDTA, and 0.1% SDS) with protease and phosphatase inhibitor cocktails (MCE, HY‐K0010, HY‐K0022, 1:100). Protein samples were denatured, subjected to SDS‐PAGE gels, transferred to PVDF membranes, blocked in 5% milk for 2 h, then immunoblotted with specific primary antibodies mentioned above. After membranes were incubated with secondary antibody, protein bands were developed by ECL developer (GE Healthcare).

### Real‐time PCR (RT‐PCR)

Total RNA was isolated by TRIzol reagent (Thermo Fisher scientific, #15596026) according to the manufacturer's instructions. Total RNA (1 µg) was used for cDNA synthesis with the PrimeScript RT reagent Kit (Takara, RR047A). RT‐PCR was then performed in triplicate or quadruplicate with a 7500 FAST RT‐PCR system (Applied Biosystems) using primers and templates that were mixed with TB Green Premix Ex Taq II kit (Takara, RR820A). The relative gene expression levels were normalized to human GAPDH and calculated using the comparative Ct (2^−ΔΔCT^) method. The specific RT‐PCR primers were listed in Table S4 (Supporting Information).

### Immunohistochemistry (IHC) and H&E Staining

ESCC tissue microarray was purchased from Shanghai Outdo Biotech Company (Shanghai, China). Clinical ESCC and adjacent tissue specimens were obtained from Linzhou Cancer Hospital (Linzhou, Henan, China) with informed consent from patients. Human subjects research in this study were approved by the Ethics Committee of China‐US (Henan) Hormel Cancer Institute (Zhengzhou, Henan, China), approval documents: CUHCIH2022002. In PDX mouse model and 4‐NQO induced ESCC mouse model, tumors or esophagi were dissected immediately after cervical dislocation, and then washed once with PBS buffer and fixed in 10% formalin or froze at ‐80 °C. Hematoxylin and eosin (H&E) staining was conducted for histopathological examination, and expression of specific proteins was detected with indicated antibodies. Imagescope software was used for scoring of IHC staining.

### MTT Assay

Briefly, cells were seeded in 96‐well plates in at least 4 replicates at a density of 1000 cells per well. The next day, MTT solution (10 µL, 5 mg mL^−1^ in PBS) was added to each well and incubated at 37 °C in 5% CO_2_ for 2 h, then DMSO (100 µL) were added after the incubation. Then absorbance was measured at 490 nm as 0‐h result. MTT assay data were collected every 24 h by the same method in the following days. Data were normalized to fold changes by 0‐h result.

### Colony Assay

Cells were seeded in 6‐well plates in triplicate at a density of 200 cells per well in 2 mL medium and cultured for 7–10 days. Then the plates were washed once by PBS buffer, stained with crystal violet solution (0.4% in ethanol) for 10 min at room temperature and then washed with water. The plates were air‐dried, and the colonies were counted.

### Soft Agar Assay

8000 cells were suspended in normal growth medium (1.5 mL) containing 0.33% agar, 1% gentamycin and glutamine (1 × 10^−3^ M). Then the mixture was immediately overlaid on solidified normal growth medium (3 mL) containing 0.5% agar, gentamycin (50 µg mL^−1^) and glutamine (1 × 10^−3^ M) in 6‐well plates. After 2‐week (KYSE450) or 3‐week (KYSE30) incubation at 37 °C in 5% CO_2_, pictures were taken through microscopy and then colonies were counted.

### Apoptosis Detection

KYSE30 and KYSE450 cells were infected with lentiviruses targeting indicated genes and scramble for 24 h, and then subjected to puromycin selection for another 48 h. Then cells were plated in 6‐well plates for treatment with DMSO or apoptosis inhibitor BOC‐D‐FMK (MCE, HY‐13229) and Z‐VAD‐FMK (MCE, HY‐16658B) at 1 × 10^−4^ M for 24 h. FITC Annexin V apoptosis detection kit with Propidium Iodide (Biolegend, #640914) was used for cell staining to determine apoptosis. Briefly, cells were collected and washed once by PBS buffer, then resuspended in Annexin V binding buffer. FITC annexin V (5 µL) and propidium iodide solution (10 µL) were added into cell suspension and mix well gently. After 15‐min incubation at room temperature in the dark, a proper volume of Annexin V binding buffer was added to each tube. Stained cells were then analyzed by flow cytometry with proper machine settings.

### Cell Cycle Analysis

KYSE30 and KYSE450 cells were infected with lentiviruses targeting indicated genes and scramble for 24 h, and then subjected to puromycin selection. After FBS starvation for 24 h, cells were cultured in complete medium with FBS for another 24 h and harvested for cell cycle analysis. Cells were washed once with PBS and fixed with cold ethanol carefully overnight. Then, after washed once with PBS, cells were permeabilized by 0.6% Triton X‐100 and treated with Rnase A (200 µg mL^−1^). Finally, cells were incubated with propidium iodide (20 µg mL^−1^) in the dark. Stained cells were then analyzed by flow cytometry with proper machine settings.

### EdU Incorporation Assay

BeyoClick EdU‐647 Cell Proliferation Kit (Beyotime, C0081L) were used to conducted the EdU incorporation assay. Briefly, after incubated with  EdU (1 × 10^−5^ m), cells were collected and fixed with 4% paraformaldehyde for 15 min, incubated with 0.3% Triton X‐100 solution for 10 min and then labelled with Alexa Fluor 647 for 30 min. The stained cells were then analyzed by flow cytometry with proper machine settings.

### Dual Luciferase Reporter Assay

Cells were transfected with pGL4.17 blank vector or pGL4.17 constructed with RTN4IP1 promoter region, as well as pRL‐TK renilla luciferase plasmid for 24 h. Then cells were lysed, and lysates were used for analysis by Dual Luciferase Reporter Assay Kit (Vazyme, DL101‐01) according to the manufacture's instruction.

### mRNA Stability

KYSE30 and KYSE450 cells were seeded in 6‐well plates. After overnight incubation, cell confluency reached ≈90%. Then, cells were treated with actinomycin D (1 µg mL^−1^) and incubated for indicated time. Cells were washed once with PBS buffer (1 mL) and collected with TRIzol reagent (0.5 mL, Invitrogen). Then, total RNA was isolated for RT‐PCR analysis.

### RNA Immunoprecipitation Assay

KYSE30 cells were lysed by RNA immunoprecipitation (RIP) buffer (1.50 × 10^−1^ M KCl, 2.5 × 10^−2^ M Tris (pH 7.4), 5 × 10^−3^ M EDTA, 1 × 10^−3^ M DTT, and 0.5% NP40) supplemented with protease inhibitor (MCE, HY‐K0010, 1:100) and RNase inhibitor (Solarbio, R8061, 1:1000). Then cell lysate was incubated with anti‐IRP2 antibody (Cell signaling technology, #37135) or normal rabbit IgG (Cell signaling technology, #2729) at 4 °C overnight. The next day, protein A/G beads (50 µL. Santa Cruz, sc‐2003) were added into the system, followed with another 4‐h incubation. Then the beads were collected by centrifugation, washed gently for 3 times with RIP buffer supplemented with RNase inhibitor. Immunoprecipitated RNA was extracted with TRIzol reagent and analyzed by RT‐PCR. Results were analyzed using the comparative Ct (2^−ΔCT^) method and then normalized with the IgG group data.

### Electrophoretic Mobility Shift Assay (EMSA)

LightShift Chemiluminescent RNA EMSA Kit (Thermo, 20158) was used, and the procedures were conducted according to the instruction. Biotin‐labeled control‐IRE probe (human FTH IRE as the positive control), biotin‐labeled RTN4IP1 IRE probes were synthesized in GenePharma, Suzhou, China. Briefly, biotin‐labeled probes were incubated with/without liver or cell cytosolic lysate for 30 min at room temperature. Then the reaction system was resolved by 5% native polyacrylamide gel, transferred to nylon membrane. Membrane was subjected to crosslinking by UV lamp for 5 min and the biotin‐labeled RNA was detected using the chemiluminescent nucleic acid Detection module kit (Thermo, 89880). Sequences of IRE probes were listed in Table S5 (Supporting Information).

### Metabolomics and Proteomic Study

KYSE450 cell were infected with lentivirus bearing scramble or RTN4IP1 shRNAs for 24 h and selected with puromycin (2 mg L^−1^) for another 48 h, essential cell passage was conducted when cell confluency reached ≈90%. 5 days after infection, cells (1×10^7^) were collected for proteomic study. The untargeted metabolomics profiling was performed on XploreMET platform (Metabo‐profile, Shanghai, China). The proteomic study was conducted by PTM Biolab (Hangzhou, China).

### Amino Acid Detection

KYSE450 cell were infected with lentivirus bearing scramble or shRNAs targeting indicated genes for 24 h and selected with 2 mg L^−1^ puromycin for another 48 h, essential cell passage was conducted when cell confluency reached ≈90%. 5 days after infection, cells (3‐5×10^6^) were collected in 4 or 5 replicate for metabolomic study. Cell was resuspended in 80% methanol (800 µL) in water, vortexed violently for 2 min and then kept at −80 °C overnight. Then samples were centrifuged at 12000 rpm for 20 min at 4 °C. Clear supernatant was collected and filtered with 0.22 µm filter. The supernatant (50 µL) was analyzed by Agilent 6460 Triple Quad LC/MS platform.

### Cell‐Derived Xenograft (CDX) Mouse Model

Animal experiments in this study were approved by the Ethics Committee of China‐US (Henan) Hormel Cancer Institute (Zhengzhou, Henan, China), approval documents: CUHCI2021032. KYSE30 (3 × 10^6^ cell per mice) or KYSE450 (1 × 10^7^ cell per mice) infected with lentivirus were subcutaneously injected into the right dorsal flank of 5‐week‐old female athymic nude mice (SPF (Beijing) Biotechnology Co., Ltd.). Tumor volume was determined at indicated time using a vernier caliper, and calculated with the formula Volume = (length) × (width)^2^ × 0.5.^[^ ^63^ ^]^ At the end, mice were sacrificed, tumors were excised and weighed.

### Patient‐Derived Xenograft (PDX) Mouse Model

For all the cancer tissues utilized in the study, written informed consent was obtained from the patients. 6‐ to 8‐week‐old female NOD‐SCID mice (Cyagen Biosciences, Suzhou, China) were used for this experiment. Tumor tissue blocks from 4th generation of LEG379 PDX model were implanted subcutaneously into mice. One week later, tumor volume was started to be measured twice a week. Once tumor volumes reached ≈200 mm^3^, tumor‐bearing mice were divided into 3 groups randomly and then injected with condensed lentivirus supernatant twice a week. Mice were sacrificed at indicated time and tumors were dissected and weighed. Then tumors were segmented and prepared for following analysis.

### 4‐NQO (4‐nitroquinoline 1‐oxide) Induced ESCC Mouse Model

The model was established as previously described.^[^ ^64^ ^]^ 8‐ to 10‐week‐old *Rtn4ip1* ^fl/fl^ and *Ed‐l2* ^cre^ *Rtn4ip1* ^fl/fl^ C57BL/6 mice were used in this experiment. Carcinogen 4‐NQO stock solution was prepared weekly, dissolved in DMSO at 100 mg mL^−1^ and diluted in autoclaved drinking water to a final concentration of 100 mg L^−1^. Mice were randomly divided into control‐group (drinking water with DMSO) and treated‐group (drinking water with 4‐NQO). Drinking water was changed once a week and mice were allowed access to drink freely. After 16‐week treatment, all drinking water was changed to normal water and maintained for another 8 weeks. Body weight data of mice was collected every 2 weeks. Mice were sacrificed at the end of the experiment and esophagi were dissected for following analysis.

### Statistics Analysis

In all statistical plots, data were expressed as the mean ± SD. Significance is indicated by ns, *p* > 0.05, *, *p <* 0.05, **, *p <* 0.01, ***, *p <* 0.001. Diversity between subgroups was assessed by student's *t*‐test/Ordinary one‐way ANOVA. Consistency between indexes was assessed by Spearman correlation coefficient analysis. ESCA tumor, ESCA normal and Esophagus–mucosa were selected to build dataset list for correlation analysis in GEPIA. Survival proportions were assessed by Log‐rank (Mantel‐Cox) test. Analysis and graphing were powered by GraphPad Prism 8.

## Conflict of Interest

The authors declare no conflict of interest.

## Author Contributions

H.F.W. designed the study, conducted the experiments, acquired data, drafted and revised the manuscript. D.Y.Z., Y.F.Z. and Q.W., acquired and analyzed data. Y.F.Z., J.M., J.L.X., T.T.L., J.Z., and P.L.W. conducted the experiments. Y.M.H., X.Y.H., M.J., and T.J.Z. participated in the discussion during the experiment. F.Q.G., D.D.Z., W.N.N., R.Y. have given the technical or material support. D.Y.Z., Y.F.Z. and K.D.L. reviewed and edited the manuscript. Z.G.D. and K.D.L. obtained the funding and supervised the study. All of the authors have read and approved the paper.

## Supporting information



Supporting Information

## Data Availability

The data that support the findings of this study are available on request from the corresponding author. The data are not publicly available due to privacy or ethical restrictions.
