# Supplementary material for: RTN4IP1 Contributes to ESCC via Regulation of Amino Acid Transporters
Source: Adv Sci (Weinh). 2025 Jan 5;12(8):2406220. doi: 10.1002/advs.202406220 (PMC11848606; doi:10.1002/advs.202406220)
Supplement: Supplementary file 1 — Supporting Information [file ADVS-12-2406220-s001.docx]

Supporting Information

RTN4IP1 contributes to ESCC via regulation of amino acid transporters

Huifang Wei, Dengyun Zhao, Yafei Zhi, Qiong Wu, Jing Ma, Jialuo Xu, Tingting Liu, Jing Zhang, Penglei Wang, Yamei Hu, Xinyu He, Fangqin Guo, Ming Jiang, Dandan Zhang, Wenna Nie, Ran Yang, Tongjin Zhao, Zigang Dong,* and Kangdong Liu*


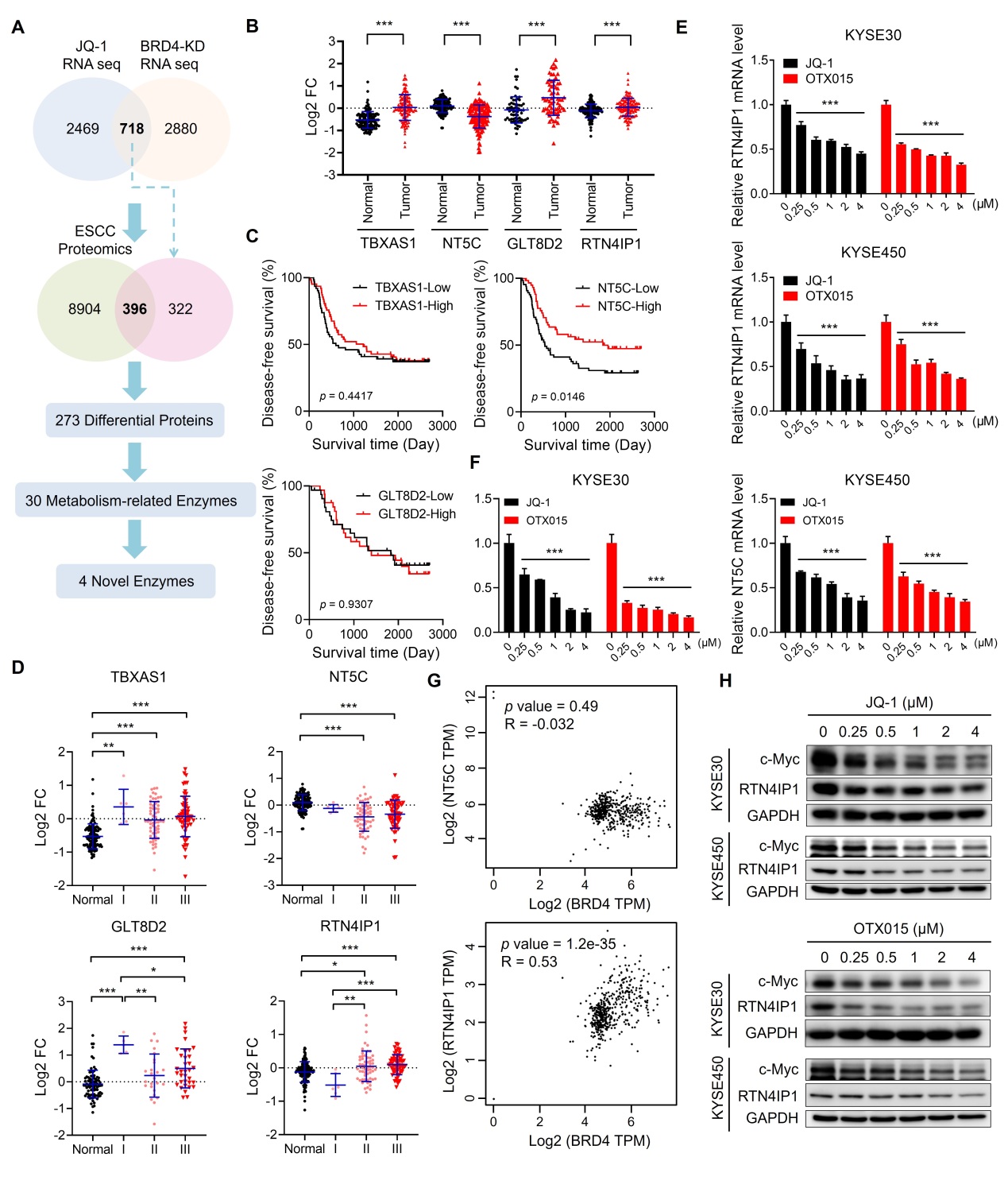


**Figure S1**. RTN4IP1 is a novel potential metabolism-related target of BRD4 in ESCC. A) Scheme displaying the procedure of screening candidates. B) Graph showing the analysis of expression patterns of candidate proteins in ESCC proteomic study. *n =*124. C) Graphs showing disease-free survival analyses of indicated proteins in ESCC proteomic study. TBXAS1-Low, *n* = 62, TBXAS1-High, *n* = 62. NT5C-Low, *n* = 65, NT5C-High, *n* = 59. GLT8D2-Low, *n* = 62, GLT8D2-High, *n* = 62. D) Graphs showing the expression of 4 candidate genes in ESCC patients with different stages. Normal, *n =*124, stage I, *n =*6, stage II, *n =*58, stage III, *n =*60. E, F) KYSE30 and KYSE450 cells treated with JQ-1 or OTX015 at indicated concentrations for 24 h were analyzed by RT-PCR. G) Graphs showing correlation analyses of *RTN4IP1*, *NT5C* and *BRD4* mRNA levels in ESCC by GEPIA. H) KYSE30 and KYSE450 cells treated with JQ-1 or OTX015 at indicated concentrations for 24 h were analyzed by Western blotting. In all statistical plots, data were expressed as the mean ± SD. *, *p <* 0.05, **, *p <* 0.01, ***, *p <* 0.001 by Student’s *t*-test (B) or Ordinary one-way ANOVA (D, E, and F). Log-rank (Mantel-Cox) test was used for comparison of survival distributions (C).


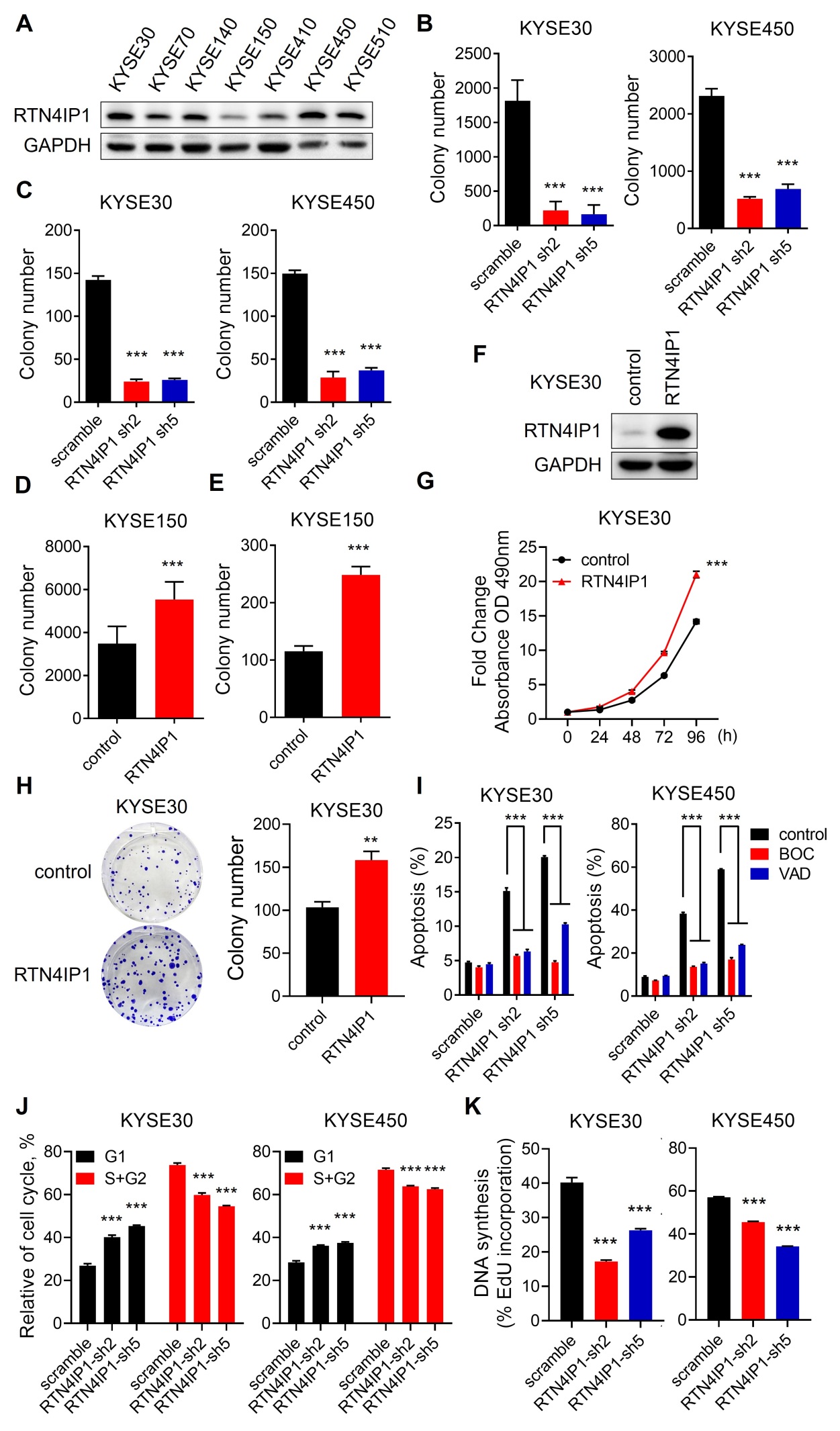


**Figure S2**. RTN4IP1 is involved in cell proliferation and survival of ESCC cells. A) Different ESCC cell lines were analyzed by Western blotting. B) Graphs showing the analysis of soft agar assay and C) colony assay of scramble and RTN4IP1-knockdown cells. D) Graphs showing the analysis of soft agar assay and E) colony assay of control and RTN4IP1-overexpressing cells. F) KYSE30 transfected with RTN4IP1 and control plasmids were analyzed by Western blotting, G) MTT assay and H) colony formation assay. I) KYSE30 and KYSE450 cells infected with lentivirus-mediated shRNAs targeting RTN4IP1 or scramble were treated with apoptosis inhibitors BOC (BOC-D-FMK), VAD (Z-VAD-FMK) or DMSO for 24 h, then analyzed by flow cytometry. J, K) KYSE30 and KYSE450 cells infected with lentivirus-mediated shRNAs targeting RTN4IP1 or scramble were analyzed by EdU incorporation and cell cycle assays. In all statistical plots, data were expressed as the mean ± SD. ***, *p <* 0.001 by Student’s *t*-test (D, E, G, and H) or Ordinary one-way ANOVA (B, C, I, J, and K).


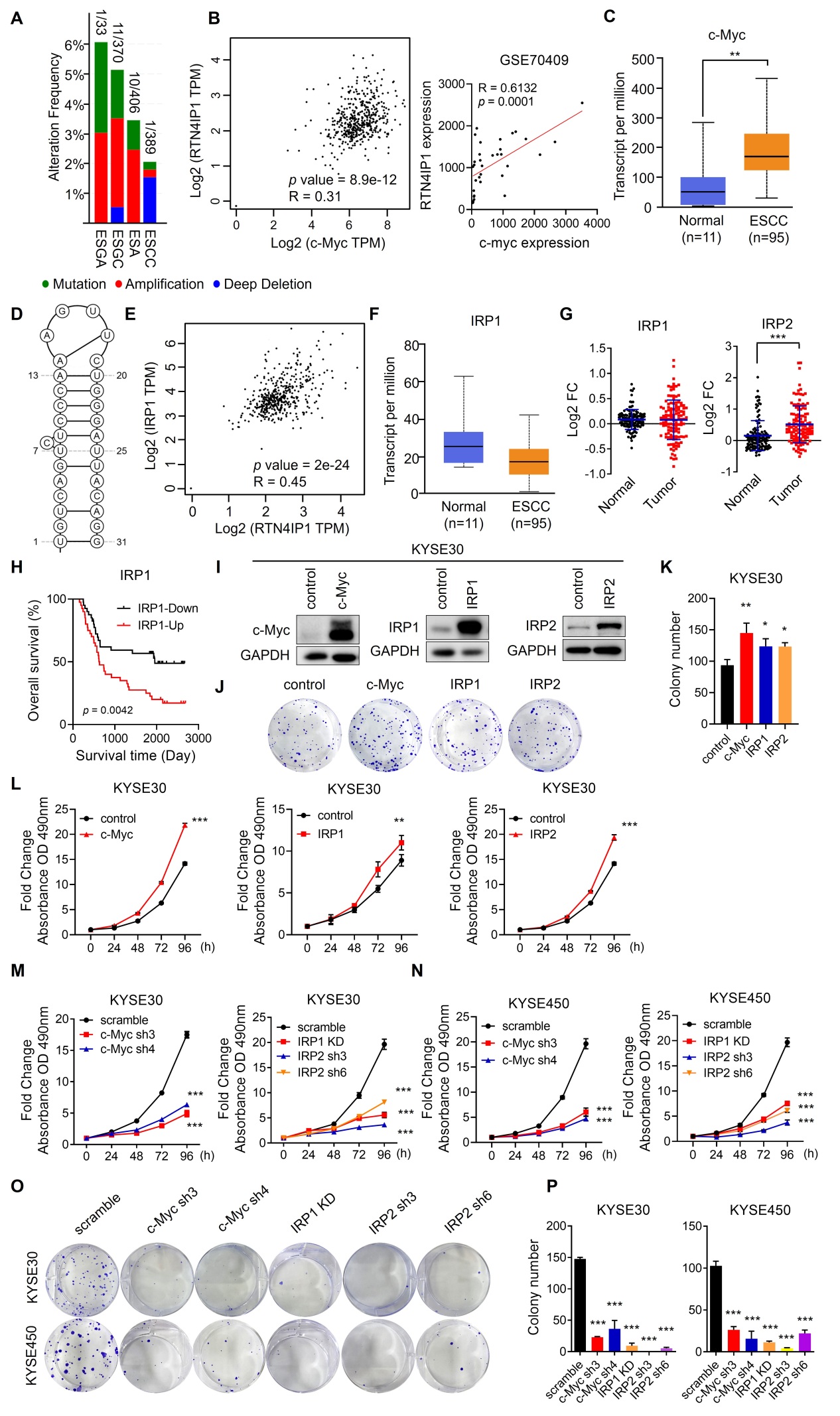


**Figure S3**. c-Myc and IRPs are involved in RTN4IP1 expression and cell proliferation in ESCC. A) RTN4IP1 gene alteration analysis in esophageal cancer by cBioPortal, number of cases with RTN4IP1 amplification and total case number were labeled. ESGA: esophagogastric adenocarcinoma. ESGC: esophagogastric cancer. ESA: esophageal adenocarcinoma. ESCC: esophageal squamous cell carcinoma. B) Graph showing correlation analysis of RTN4IP1 and c-Myc in ESCC analyzed by GEPIA (left) and GEO dataset (right). C) Graph showing mRNA expression pattern of c-Myc in ESCC and normal esophageal tissues analyzed by UALCAN, *p =* 0.0074. D) Schematic showing the IRE in RTN4IP1 mRNA 3’UTR. E) Graph showing correlation analysis of RTN4IP1 and IRP1 in ESCC by GEPIA. F) Graph showing mRNA expression pattern of IRP1 in ESCC and normal esophageal tissues analyzed by UALCAN, *p =* 0.0806. G) Graphs showing analysis of IRP1 and IRP2 expression patterns in ESCC proteomic study, *n =*124 pairs. H) Graph showing the overall survival analysis of IRP1 in ESCC proteomic study. IRP1-Up, *n* = 40, IRP1-Down, *n* = 40. I-L) KYSE30 cell were transfected with control, c-Myc, IRP1 or IRP2 plasmids and analyzed by Western blotting (I), colony formation assay (J, K) and MTT assay (L). M-P) KYSE30 and KYSE450 cells infected with lentivirus-mediated shRNAs targeting c-Myc, IRP1, IRP2 or scramble were analyzed by MTT assay (M, N) and colony formation assay (O, P). In all statistical plots, data were expressed as the mean ± SD. **, *p <* 0.01, ***, *p <* 0.001 by Student’s *t*-test (G and L) or Ordinary one-way ANOVA (K, M, N, and P).


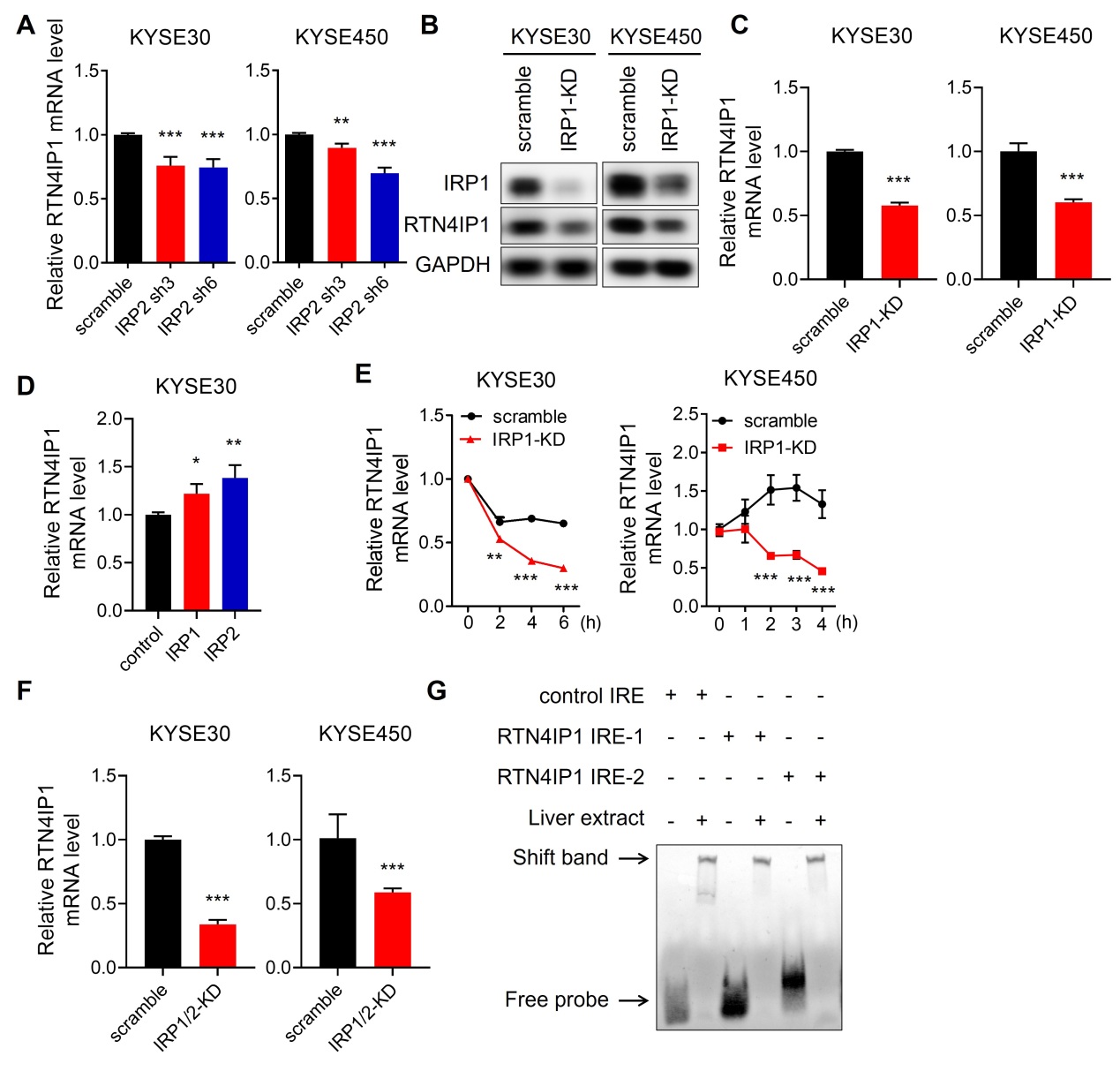


**Figure S4**. IRP1/2 regulates RTN4IP1 mRNA stability. A) KYSE30 and KYSE450 cells infected with lentivirus-mediated shRNAs targeting IRP2 or scramble were analyzed by RT-PCR. B-C) KYSE30 and KYSE450 cells infected with lentivirus-mediated shRNAs targeting IRP1 or scramble were analyzed by Western blotting and RT-PCR. D) KYSE30 cell transfected with control, IRP1 or IRP2 plasmids were analyzed by RT-PCR. E) KYSE30 and KYSE450 cells infected with lentivirus-mediated shRNAs targeting IRP1 or scramble were treated with actinomycin D for indicated time and then analyzed by RT-PCR. F) KYSE30 and KYSE450 cells infected with lentivirus-mediated shRNAs targeting IRP1/2 or scramble were analyzed by RT-PCR. G) Interaction between IRPs and indicated IRE probes were determined by EMSA. Biotin-labeled IRE Probes were incubated with liver cytosolic lysate. In all statistical plots, data were expressed as the mean ± SD. *, *p <* 0.05, **, *p <* 0.01, ***, *p <* 0.001 by Student’s *t*-test (C, E, and F) or Ordinary one-way ANOVA (A and D).


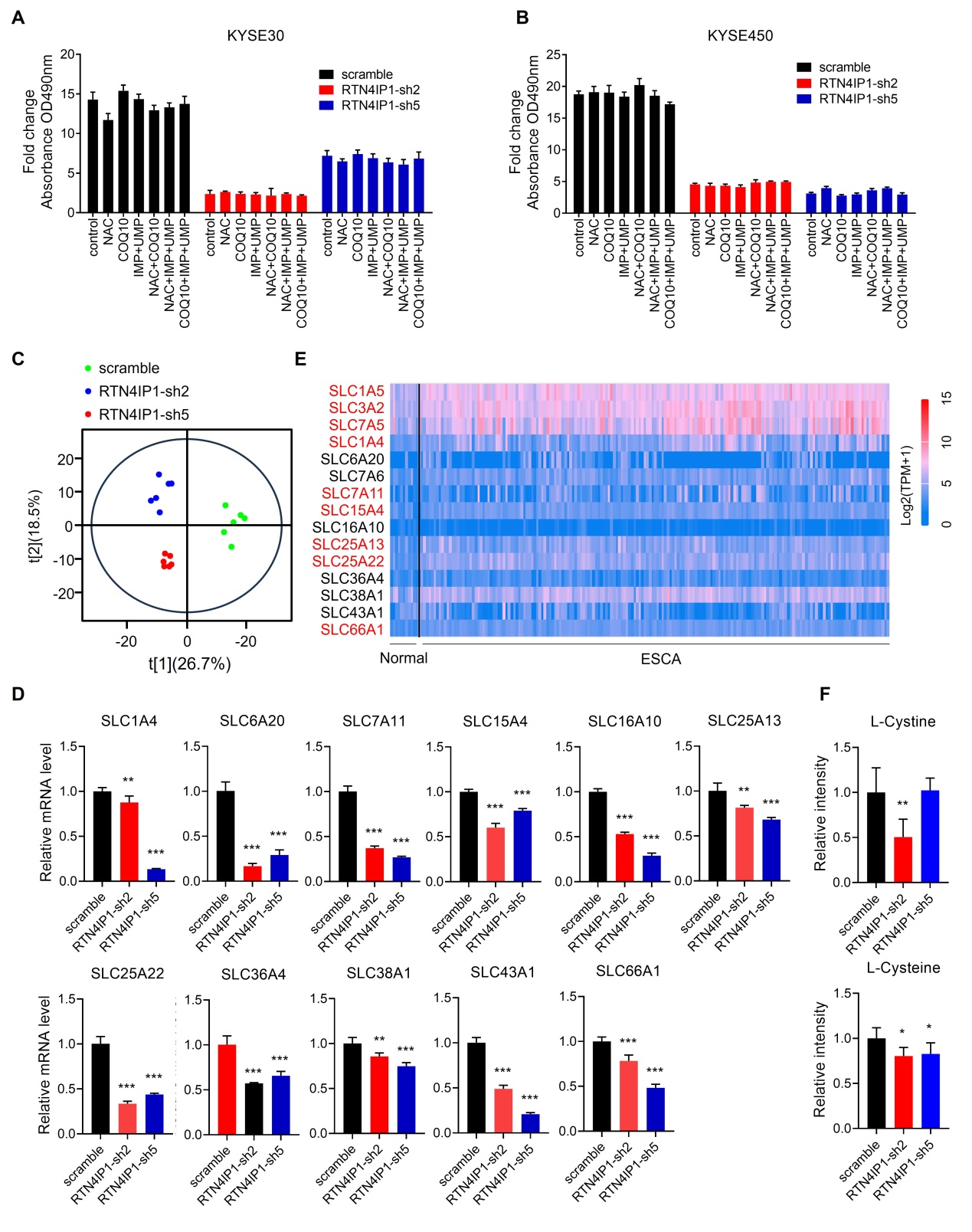


**Figure S5**. RTN4IP1 deficiency leads to downregulation of multiple amino acid transporters. A) KYSE30 and B) KYSE450 cells infected with lentivirus-mediated shRNAs targeting RTN4IP1 or scramble for 48 h were supplied with indicated compounds, and cell viability were determined by MTT assay. Graphs showing the fold change of absorbance at 72 h after compounds addition. C) The scoring plot of OPLS-DA model of the metabolomics study. D) KYSE450 cells infected with lentivirus-mediated shRNAs targeting RTN4IP1 or scramble were analyzed by RT-PCR. Graphs showing relative mRNA levels of indicated genes. E) Graph showing the expression patterns of indicated amino acid transporters in ESCA from UALCAN database. Amino acid transporters marked in red are significantly upregulated in ESCC (*p <* 0.01). F) Graphs showing relative levels of L-Cystine and L-Cysteine in RTN4IP1 knockdown or scramble KYSE450 cells. In all statistical plots, data were expressed as the mean ± SD. *, *p <* 0.05, **, *p <* 0.01, ***, *p <* 0.001 by Ordinary one-way ANOVA (D and F).


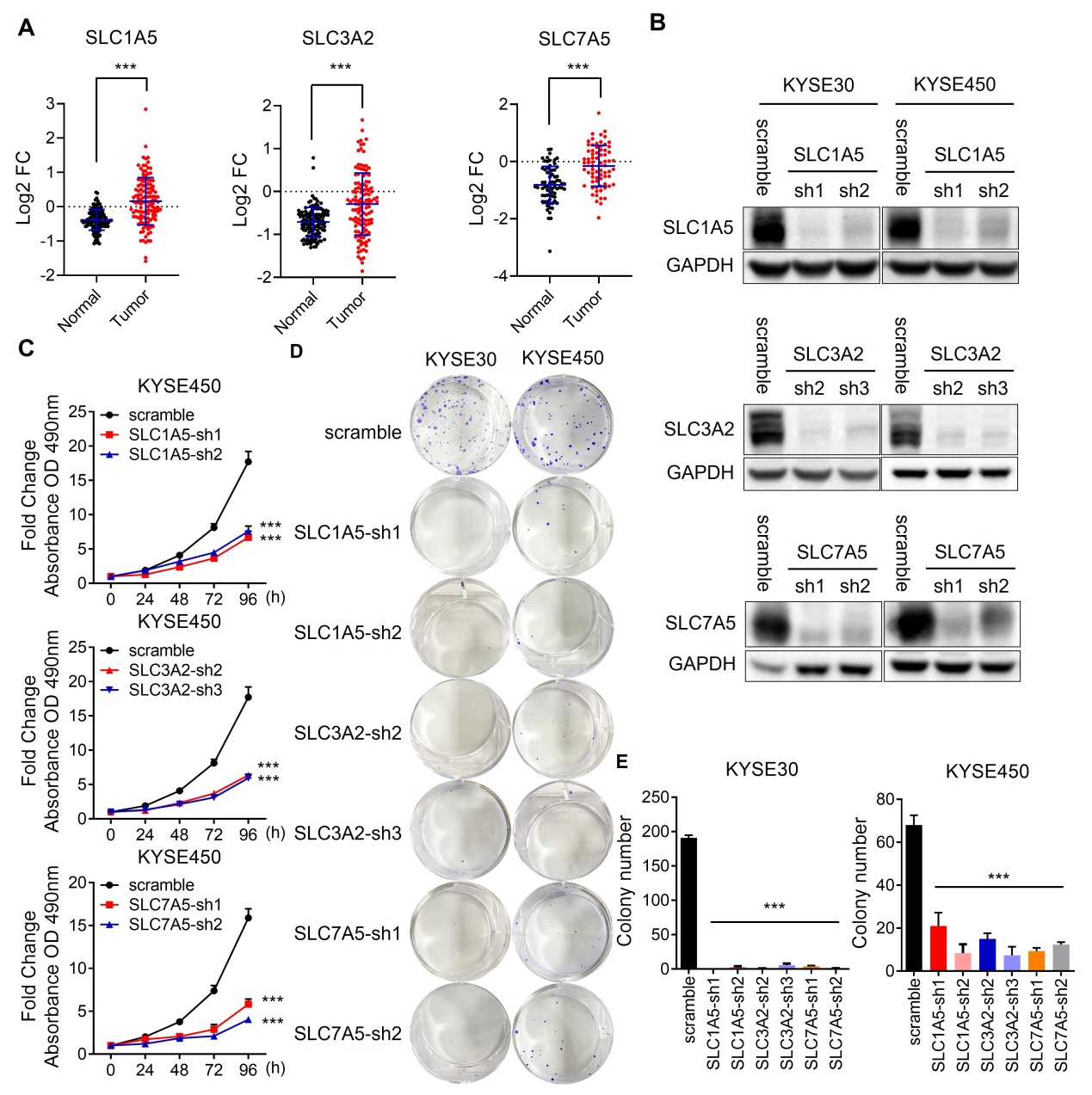


**Figure S6.** SLC1A5, SLC3A2, and SLC7A5 are essential to cell proliferation. A) Graphs showing analysis of expression patterns of indicated proteins in ESCC proteomic study. SLC1A5 and SLC3A2, *n =*124 pairs; SLC7A5, *n =*60 pairs. B-E) KYSE30 and KYSE450 cells infected with lentivirus-mediated shRNAs targeting SLC1A5, SLC3A2, SLC7A5 or scramble were analyzed by Western blotting (B), MTT assay (C) and colony assay (D and E). In all statistical plots, data were expressed as the mean ± SD. ***, *p <* 0.001 by Student’s *t*-test (A) or Ordinary one-way ANOVA (C and E).


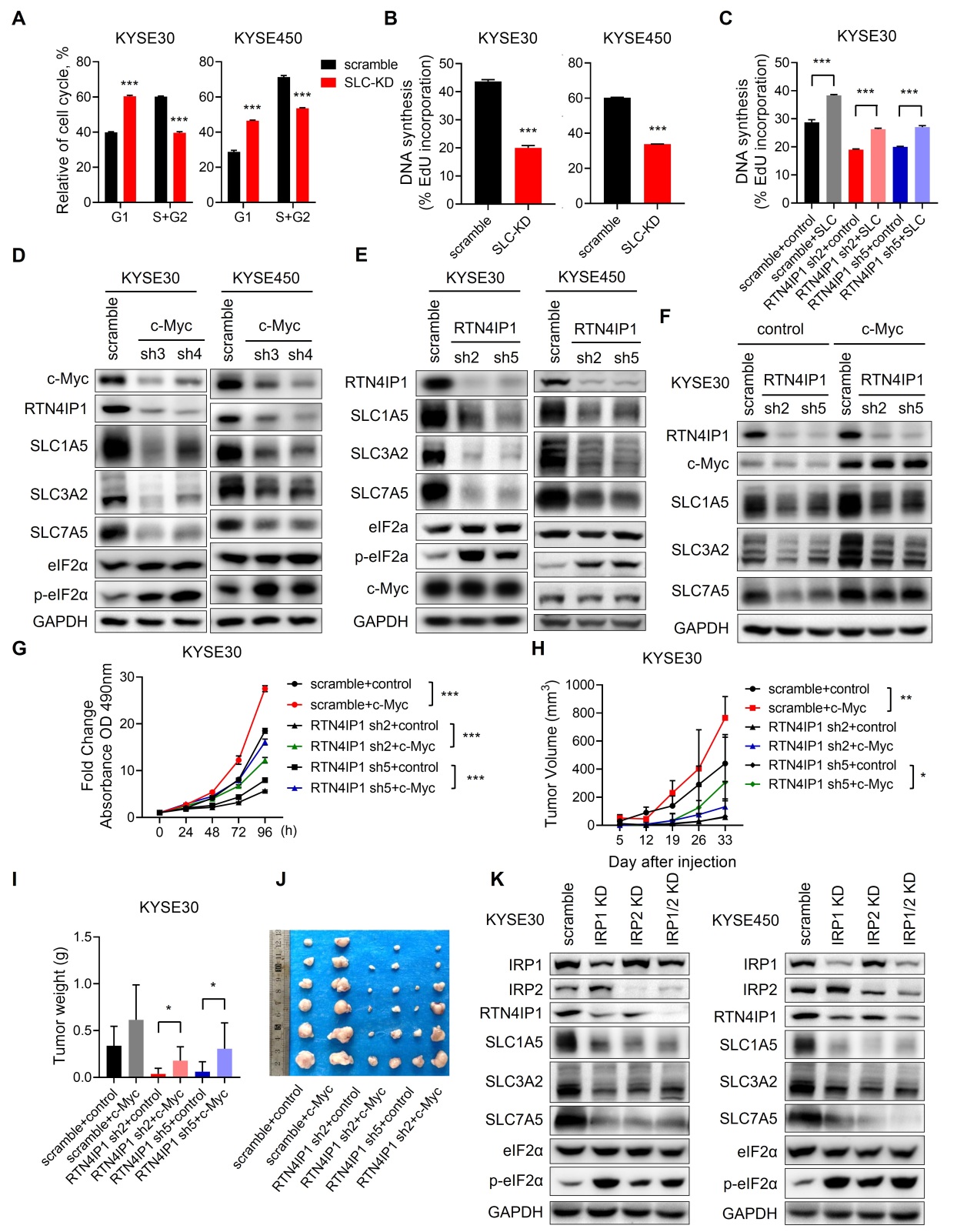


**Figure S7**. SLC1A5, SL3A2, and SLC7A5 are essential to RTN4IP1-mediated cell proliferation in ESCC. A, B) KYSE30 and KYSE450 cells infected with lentivirus-mediated shRNAs targeting SLC1A5/SLC3A2/SLC7A5 or scramble were analyzed by cell cycle (A) and EdU incorporation assays (B). C) KYSE30 cells infected with lentivirus-mediated shRNAs targeting RTN4IP1 or scramble were transfected with lentivirus-mediated overexpression of control or SLC1A5/SLC3A2/SLC7A5 and then analyzed by EdU incorporation assay. D) KYSE30 and KYSE450 cells infected with lentivirus-mediated shRNAs targeting c-Myc or scramble were analyzed by Western blotting. E) KYSE30 and KYSE450 cells infected with lentivirus-mediated shRNAs targeting RTN4IP1 or scramble were analyzed by Western blotting. F,G) KYSE30 cells infected with lentivirus-mediated shRNAs targeting RTN4IP1 or scramble were transfected with c-Myc or control plasmids and then analyzed by Western blotting (F) and MTT (G). H-J) Growth curves (H) and weight data (I) of xenograft tumors derived from indicted KYSE30 cells. Volumes of xenograft tumors were measured at indicated time and showed statistically. Images showed xenograft tumors (J). K) KYSE30 and KYSE450 cells co-infected with lentivirus-mediated shRNAs targeting IRP1/2 or scramble were analyzed by Western blotting. In all statistical plots, data were expressed as the mean ± SD. *, *p <* 0.05, **, *p <* 0.01, ***, *p <* 0.001 by Student’s *t*-test (A, B, H, and I) or Ordinary one-way ANOVA (C and G).


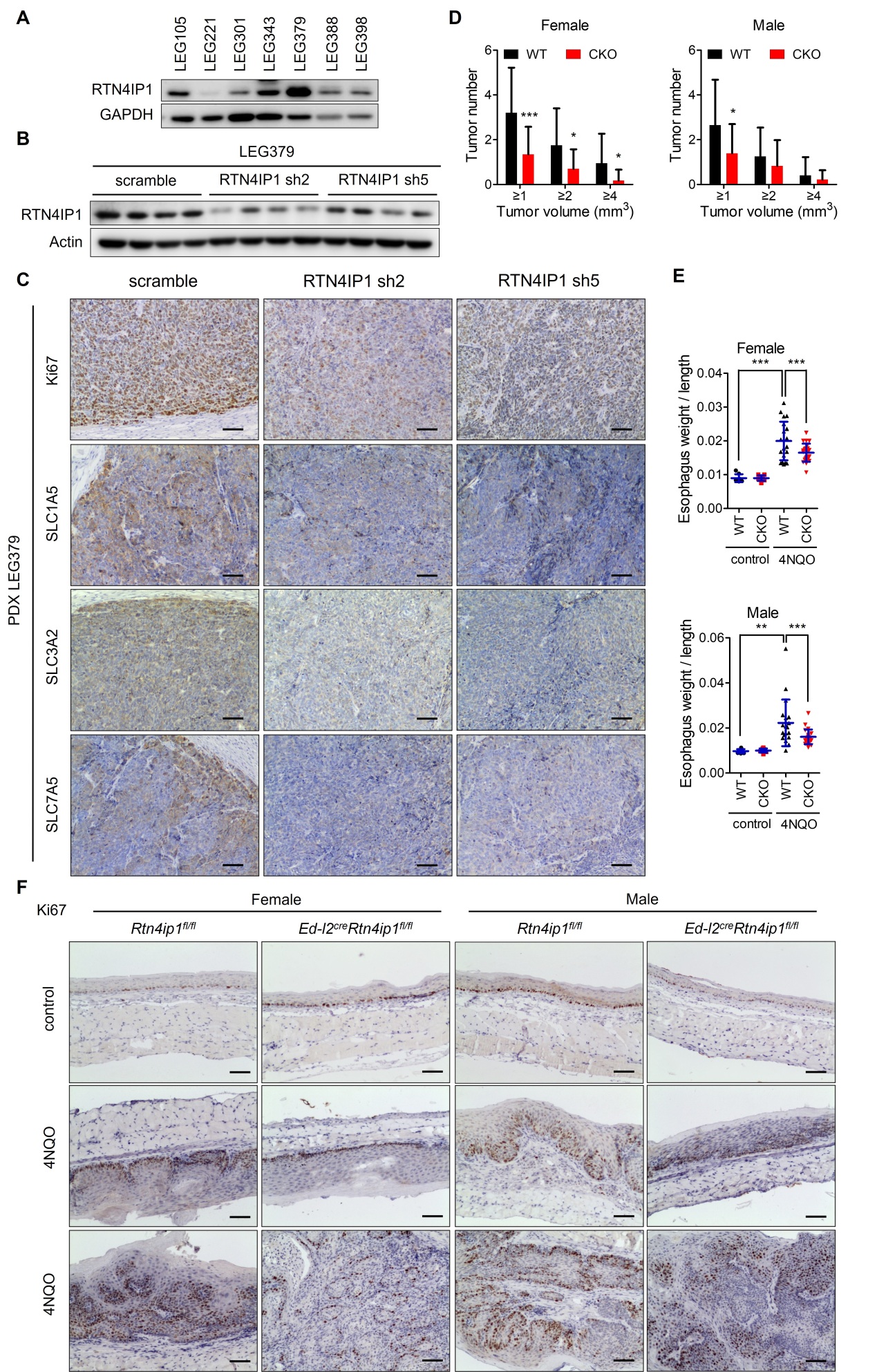


**Figure S8**. RTN4IP1 deficiency retards tumor growth in ESCC. A) PDX tissues from different ESCC cases were analyzed by Western blotting. B) Tumors derived from LEG379 PDX model after lentivirus injection were analyzed by Western blotting. C) Representative images of Ki67, SLC1A5, SLC3A2, and SLC7A5 staining of tumors derived from LEG379 PDX model after lentivirus injection. Scale bar = 100 μm. D) Graphs showing analysis of tumor volume distribution of indicated groups in 4-NQO treated *Rtn4ip1*^fl/fl^ mice (WT) and *Ed-l2*^cre^ *Rtn4ip1*^fl/fl^ mice (CKO). E) Graphs showing the analysis of the weight/length ratio of esophagi in indicated groups. F) Representative images of Ki67 staining of esophagi from *Rtn4ip1*^fl/fl^ mice and *Ed-l2*^cre^ *Rtn4ip1*^fl/fl^ mice in 4NQO and control groups. Scale bar = 100 μm. In all statistical plots, data were expressed as the mean ± SD. *, *p <* 0.05, **, *p <* 0.01, ***, *p <* 0.001 by Student’s *t*-test (D) or Ordinary one-way ANOVA (E).


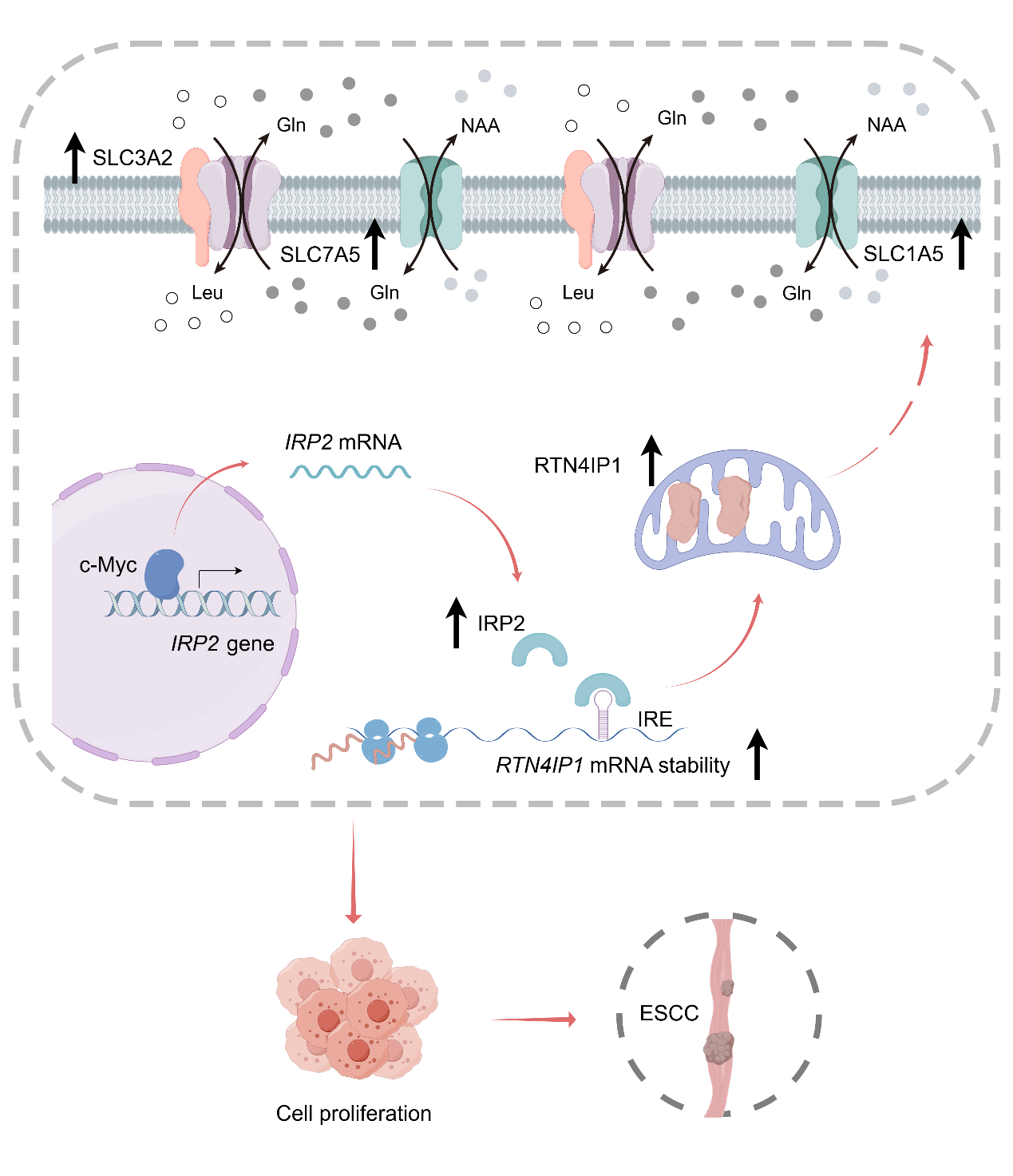


**Figure S9**. Schematic illustration showing the role of amino acid uptake mediated by c-Myc‑IRP2‑RTN4IP1 axis in ESCC carcinogenesis. Gln, glutamine. Leu, leucine. NAA, natural amino acids.

**Table S1.** Spearman correlation coefficient analysis of RTN4IP1 and amino acid transporters.

| Gene | R value | *p* value | Gene | R value | *p* value |
| --- | --- | --- | --- | --- | --- |
| *SLC25A15* | 0.71 | 4.50E-74 | *SLC6A19* | 0.29 | 2.70E-10 |
| *SLC25A13* | 0.7 | 5.50E-71 | *SLC43A1* | 0.28 | 6.10E-10 |
| *SLC38A1* | 0.62 | 1.00E-50 | *SLC7A5* | 0.26 | 7.30E-09 |
| *SLC38A7* | 0.61 | 7.40E-50 | *SLC6A5* | 0.24 | 1.00E-07 |
| *SLC1A4* | 0.59 | 9.20E-45 | *SLC1A2* | 0.22 | 2.50E-06 |
| *SLC25A22* | 0.57 | 1.90E-41 | *SLC25A12* | 0.18 | 7.80E-05 |
| *SLC7A7* | 0.54 | 1.20E-36 | *SLC38A5* | 0.17 | 3.10E-04 |
| *SLC16A10* | 0.52 | 2.10E-33 | *SLC7A10* | 0.17 | 1.60E-04 |
| *SLC66A1* | 0.51 | 4.10E-32 | *SLC7A9* | 0.11 | 0.023 |
| *SLC43A2* | 0.5 | 1.30E-46 | *SLC7A1* | 0.089 | 0.053 |
| *SLC36A4* | 0.49 | 2.90E-30 | *SLC38A2* | 0.071 | 0.13 |
| *SLC38A9* | 0.48 | 5.90E-29 | *SLC25A18* | 0.066 | 0.15 |
| *SLC7A11* | 0.48 | 3.20E-28 | *SLC17A6* | 0.064 | 0.17 |
| *SLC25A38* | 0.47 | 3.70E-27 | *SLC25A2* | 0.056 | 0.065 |
| *SLC1A5* | 0.46 | 4.30E-26 | *SLC36A2* | 0.047 | 0.31 |
| *SLC15A4* | 0.44 | 2.50E-23 | *SLC7A13* | 0.028 | 0.54 |
| *SLC66A4* | 0.44 | 2.80E-23 | *SLC32A1* | 0.0098 | 0.83 |
| *SLC6A20* | 0.44 | 1.90E-23 | *SLC1A6* | 0.0067 | 0.88 |
| *SLC3A2* | 0.42 | 4.00E-21 | *SLC6A15* | -0.0034 | 0.94 |
| *SLC36A1* | 0.4 | 7.70E-20 | *SLC38A4* | -0.032 | 0.49 |
| *SLC1A1* | 0.39 | 2.50E-18 | *SLC6A17* | -0.035 | 0.45 |
| *SLC7A6* | 0.39 | 4.80E-18 | *SLC7A2* | -0.042 | 0.36 |
| *SLC38A10* | 0.38 | 8.30E-18 | *SLC1A3* | -0.048 | 0.3 |
| *SLC6A7* | 0.38 | 3.30E-17 | *SLC7A14* | -0.048 | 0.3 |
| *SLC6A14* | 0.35 | 1.20E-14 | *SLC7A8* | -0.12 | 0.012 |
| *SLC3A1* | 0.32 | 1.00E-12 | *SLC38A3* | -0.13 | 0.0052 |
| *SLC15A3* | 0.31 | 3.50E-12 | *SLC6A9* | -0.14 | 0.0024 |
| *SLC17A8* | 0.3 | 3.60E-11 | *SLC1A7* | -0.16 | 0.00048 |
| *SLC38A8* | 0.29 | 1.60E-10 | *SLC7A3* | -0.16 | 0.00072 |
| *SLC6A18* | 0.29 | 9.20E-11 | *SLC17A7* | -0.35 | 8.30E-15 |

**Table S2.** Primers for gene clone.

| Gene | NCBI RefSeq | Primer | Vector |
| --- | --- | --- | --- |
| *RTN4IP1* | NM_032730.5 | Forward: ATGGAATTTCTGAAGACTTGT | pCDNA4-TO-MYC-His B |
|  |  | Reverse: AACAACATTAATTACAGTCTTTCC |  |
| *c-Myc* | NM_001354870.1 | Forward: ATGGATTTTTTTCGGGTAGTGGAAA | pCDNA4-TO-MYC-His B |
|  |  | Reverse: CGCACAAGAGTTCCGTAGCTGTT | pCDNA3.1-EGFP |
| *IRP1* | NM_001278352.2 | Forward: ATGAGCAACCCATTCGCACACCTT | pCDNA3.1-3×flag |
|  |  | Reverse: CTACTTGGCCATCTTGCGGATCAT |  |
| *IRP2* | NM_004136.4 | Forward: ATGGACGCCCCAAAAGCAGGATAC | pCDNA3.1-3×flag |
|  |  | Reverse: TGAGAATTTTCGTGCCACAAAG |  |
| *RTN4IP1* promoter |  | Forward: CACAGCAGGTGATTACAGTCAACAA | pGL4.17 [*luc2*/Neo] |
|  |  | Reverse: AGTATTCTGTCCATTCTCCTCCCTC |  |

**Table S3.** shRNA primers for knockdown

| shRNA | Primers |
| --- | --- |
| RTN4IP1 sh2 | Forward: CCGGGTCCATTATCGCTGGGCATTTCTCGAGAAATGCCCAGCGATAATGGACTTTTTG |
|  | Reverse: AATTCAAAAAGTCCATTATCGCTGGGCATTTCTCGAGAAATGCCCAGCGATAATGGAC |
| RTN4IP1 sh5 | Forward: CCGGCAAGGCACTCTTTCAGAGTTTCTCGAGAAACTCTGAAAGAGTGCCTTGTTTTTG |
|  | Reverse: AATTCAAAAACAAGGCACTCTTTCAGAGTTTCTCGAGAAACTCTGAAAGAGTGCCTTG |
| c-Myc sh3 | Forward: CCGGGAACTATGACCTCGACTACGACTCGAGTCGTAGTCGAGGTCATAGTTCTTTTTG |
|  | Reverse: AATTCAAAAAGAACTATGACCTCGACTACGACTCGAGTCGTAGTCGAGGTCATAGTTC |
| c-Myc sh4 | Forward: CCGGCCCAAGGTAGTTATCCTTAAACTCGAGTTTAAGGATAACTACCTTGGGTTTTTG |
|  | Reverse: AATTCAAAAACCCAAGGTAGTTATCCTTAAACTCGAGTTTAAGGATAACTACCTTGGG |
| IRP1 sh | Forward: CCGGCGTGTATTACACCAGTGTTAACTCGAGTTAACACTGGTGTAATACACGTTTTTG |
|  | Reverse: AATTCAAAAACGTGTATTACACCAGTGTTAACTCGAGTTAACACTGGTGTAATACACG |
| IRP2 sh3 | Forward: CCGGGAAGGTATCCCACTGATTATTCTCGAGAATAATCAGTGGGATACCTTCTTTTTG |
|  | Reverse: AATTCAAAAAGAAGGTATCCCACTGATTATTCTCGAGAATAATCAGTGGGATACCTTC |
| IRP2 sh6 | Forward: CCGGCCTCAGTTCAAGTGGAGTATTCTCGAGAATACTCCACTTGAACTGAGGTTTTTG |
|  | Reverse: AATTCAAAAACCTCAGTTCAAGTGGAGTATTCTCGAGAATACTCCACTTGAACTGAGG |
| SLC1A5 sh1 | Forward: CCGGGCAGTCCTTGGACTTCGTAAACTCGAGTTTACGAAGTCCAAGGACTGCTTTTTG |
|  | Reverse: AATTCAAAAAGCAGTCCTTGGACTTCGTAAACTCGAGTTTACGAAGTCCAAGGACTGC |
| SLC1A5 sh2 | Forward: CCGGGCTGCTTATCCGCTTCTTCAACTCGAGTTGAAGAAGCGGATAAGCAGCTTTTTG |
|  | Reverse: AATTCAAAAAGCTGCTTATCCGCTTCTTCAACTCGAGTTGAAGAAGCGGATAAGCAGC |
| SLC3A2 sh2 | Forward: CCGGAGTCTCTTGCAATCGGCTAAACTCGAGTTTAGCCGATTGCAAGAGACTTTTTTG |
|  | Reverse: AATTCAAAAAAGTCTCTTGCAATCGGCTAAACTCGAGTTTAGCCGATTGCAAGAGACT |
| SLC3A2 sh3 | Forward: CCGGGCCTACTCGAATCCAACAAAGCTCGAGCTTTGTTGGATTCGAGTAGGCTTTTTG |
|  | Reverse: AATTCAAAAAGCCTACTCGAATCCAACAAAGCTCGAGCTTTGTTGGATTCGAGTAGGC |
| SLC7A5 sh1 | Forward: CCGGCTAGATCCCAACTTCTCATTTCTCGAGAAATGAGAAGTTGGGATCTAGTTTTTG |
|  | Reverse: AATTCAAAAACTAGATCCCAACTTCTCATTTCTCGAGAAATGAGAAGTTGGGATCTAG |
| SLC7A5 sh2 | Forward: CCGGGCATTATACAGCGGCCTCTTTCTCGAGAAAGAGGCCGCTGTATAATGCTTTTTG |
|  | Reverse: AATTCAAAAAGCATTATACAGCGGCCTCTTTCTCGAGAAAGAGGCCGCTGTATAATGC |

**Table S4.** Primers for RT-PCR and mouse genotyping

| Gene | Primer |
| --- | --- |
| RTN4IP1 | Forward: GGAGTCACTGTAGGTTCAAAGGC |
|  | Reverse: CTTTCCCGCATCCACCAGTTCT |
| GAPDH | Forward: TGCACCACCAACTGCTTAGC |
|  | Reverse: TCTTCTGGGTGGCAGTGATG |
| SLC1A5 | Forward: TCCTCTTCACCCGCAAAAACCC |
|  | Reverse: CCACGCCATTATTCTCCTCCAC |
| SLC3A2 | Forward: CCAGAAGGATGATGTCGCTCAG |
|  | Reverse: GAGTAAGGTCCAGAATGACACGG |
| SLC7A5 | Forward: GCCACAGAAAGCCTGAGCTTGA |
|  | Reverse: ATGGTGAAGCCGATGCCACACT |
| SLC1A4 | Forward: TGTGGTTGCAGCTTTCCGTACG |
|  | Reverse: CCAGAGCAAACAGGACCAATCC |
| SLC6A20 | Forward: CAGCGAGATGTTCCCGCAAATC |
|  | Reverse: GCCTCTGTGTAGACGATGAATGC |
| SLC7A11 | Forward: TCCTGCTTTGGCTCCATGAACG |
|  | Reverse: AGAGGAGTGTGCTTGCGGACAT |
| SLC15A4 | Forward: CCTCTGAAGGACAAACTGGTCG |
|  | Reverse: ACAAGGTTCAGCCTTTTACTCTCC |
| SLC16A10 | Forward: GGATACTTTGTGCCTTATGTTCAC |
|  | Reverse: AAGAGCAGTCGTCCAACTCCTG |
| SLC25A13 | Forward: AGATGGTTCGGTCCCACTTGCA |
|  | Reverse: ACCAGTGGTGATTTCTCCTGCC |
| SLC25A22 | Forward: GTCAACGAGGACACCTACTCTG |
|  | Reverse: GGAAGTAGACCACCTGTGCGAT |
| SLC36A4 | Forward: GCAAGAGCTTCTGCCTGTTCAG |
|  | Reverse: TGCCAATGGAAGTCCTAAAAGGC |
| SLC38A1 | Forward: CTTTGGAGCCACCTCTCTACAG |
|  | Reverse: ACCAGGCTGAAAATGTCTCTTCC |
| SLC43A1 | Forward: GATGCTGGAGTACCTTGTGACTG |
|  | Reverse: CAGGTGAGAAGGCACAACAGCT |
| SLC66A1 | Forward: CTACAAGACGGGCAACATGGAC |
|  | Reverse: TCAGCATCACCAGGTCTGCCAA |
| *Rtn4ip1*^fl/fl^ | CKO-Forward: GAGTACCCACCTGCCAGCCTTTGATA |
|  | CKO-Reverse: CAAGCAAGCCTCACAGAGATCTGTC |
|  | WT-Forward: GAGTACCCACCTGCCAGCCTTTGTCC |
|  | WT-Reverse: CAAGCAAGCCTCACAGAGATCTGTC |
| *Ed-l2*^cre^ | Forward: AATGTTAATCCATATTGGCAGAACG |
|  | Reverse: TGCATGATCTCCGGTATTGAAACT |

**Table S5**. RNA probes used in EMSA assay

| IRE | Sequence |
| --- | --- |
| Biotinylated Control IRE | UCCUGCUUCAACAGUGCUUGGACGGAAC-Biotin |
| Biotinylated RTN4IP1 IRE-1 | UGUCAGUCUCCCAAAGUUCUGGGAUUACAGG-Biotin |
| Biotinylated RTN4IP1 IRE-2 | CAUUUUUCAUGGCCAGUGGCCCAUGUUUAGA-Biotin |
